# Demography and Population Dynamics of a Small Mammal Assemblage in Chilean Semiarid Thorn‐Scrub Habitat: A 30‐Year Study

**DOI:** 10.1002/ece3.72248

**Published:** 2025-10-29

**Authors:** Douglas A. Kelt, Peter L. Meserve, Alejandra J. Troncoso, W. Bryan Milstead, M. Andrea Previtali, Julio R. Gutiérrez, Madan K. Oli

**Affiliations:** ^1^ Department of Wildlife, Fish, & Conservation Biology University of California Davis California USA; ^2^ Department of Biological Sciences University of Idaho Moscow Idaho USA; ^3^ Departamento de Biología Universidad de La Serena La Serena Chile; ^4^ Centro de Estudios Avanzados en Zonas Arida (CEAZA) La Serena Chile; ^5^ Instituto de Ecología y Biodiversidad (IEB) Santiago Chile; ^6^ U.S. Environmental Protection Agency National Health and Environmental Effects Laboratory, Atlantic Ecology Division Narragansett Rhode Island USA; ^7^ Departamento de Ciencias Naturales Universidad Nacional del Litoral Santa Fe Argentina; ^8^ Department of Wildlife Ecology and Conservation University of Florida Gainesville Florida USA

**Keywords:** *Abrocoma bennettii*, *Abrothrix longipilis*, *Abrothrix olivacea*, El Niño southern oscillation, *Octodon degus*, *Oligoryzomys longicaudatus*, *Phyllotis darwini*, population ecology, seasonality, *Thylamys elegans*

## Abstract

Characterizing population dynamics in heterogeneous environments requires comprehensive long‐term data. We monitored seven small mammals in replicated sites in a semiarid Chilean thorn‐scrub habitat over 30 years using monthly capture–mark–recapture (CMR) sampling. We applied a superpopulation CMR modeling framework to examine the following: (i) How do population sizes and demographic parameters vary seasonally and over time? and (ii) Are there commonalities in the variation of those parameters either seasonally or annually? Capture probabilities among four “core” species ( *Octodon degus* , *Phyllotis darwini* , *Abrothrix olivacea*, and *Thylamys elegans* ) varied strongly over time, as did apparent survival among years and rainfall seasons, with individuals generally experiencing higher survival during the wet season. Recruitment measures also showed strong annual and seasonal variation, with higher numbers in wet seasons and years. Capture probability in three “opportunistic” species ( *Abrocoma bennettii* , *Abrothrix longipilis* , and *Oligoryzomys longicaudatus* ) varied over time, as did survival and recruitment across rainfall or reproductive seasons. As predicted, annual and seasonal variation in rainfall strongly influenced the survival and recruitment of most species, and their populations increased rapidly following rainfall events. Unsurprisingly, core species shared similar overall responses to environmental drivers; opportunistic species responded differently to seasonal or annual variation in rainfall, perhaps reflecting their origins in non‐thorn‐scrub habitat. Finally, for all species, population size correlated more strongly with the number of recruits than with survival, suggesting that the former has a greater influence on the dynamics of our study populations. This study provides the first insight into the demography of the entire small mammal community at our study site, and in particular, the demography of *A. bennettii* , *A. longipilis* , and *O. longicaudatus* from semiarid habitat. Our results, based on the longest time series in South America, provide comprehensive demographic information on a diverse small mammal community, and offer novel insight into community‐level response to changing climate.

## Graphical abstract

Using the longest and most comprehensive demographic database available in temperate South America, we characterize the population dynamics of an entire community of small mammals in a semiarid environment in north‐central Chile using a superpopulation CMR modeling framework. Abiotic drivers—annual and seasonal rainfall—are the strongest influences on survival and recruitment, and core species (those present in effectively all monthly surveys over 30 years) share similar responses to environmental drivers. In contrast, opportunistic species, which appear to favor more mesic habitats that occur near our core study area and therefore disappear for months to years during dry periods, responded individualistically to seasonal or annual variation in rainfall. For all species, population size correlated more strongly with the number of recruits than with survival, suggesting that the former has a greater influence on the dynamics of our study populations.

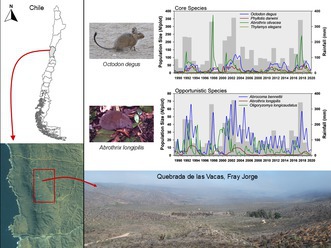

## 1 Introduction

Understanding the distribution and abundance of organisms remains a fundamental goal of ecology; more than half a century ago, this was the focus of two classic treatises (Andrewartha and Birch 1954; Lack 1954), and it continues to drive much ecological research today. Small mammals are ideal subjects for understanding population regulation, as their relatively short life spans and relatively rapid reproductive rates (e.g., generally, they are r‐selected) mean that entire generations can be tracked over relatively brief periods of time. Additionally, some species exhibit relatively regular cycles in abundance, which has led to an enormous literature on factors regulating both quantitative (e.g., cycle length, amplitude) and qualitative (e.g., behavior, body size, physiology) aspects of cycle phase (Krebs 2013; Oli 2019). However, the populations of most small mammals do not cycle regularly; instead, their population dynamics are driven primarily by relatively episodic extrinsic factors (e.g., rainfall), with density‐dependent effects playing secondary roles. These are the “inextricably intertwined endogenous and exogenous forces” that Lima et al. (1999) argue are the sources of rodent outbreaks in western South America. Unfortunately, most research efforts extend only a few years, making it challenging to fully capture the range of dynamics and influences driving small mammal population dynamics. Numerous authors have highlighted the value and need for further long‐term studies (e.g., Cody and Smallwood 1996; Hayes and Schradin 2017; Hughes et al. 2017; Jones and Driscoll 2022; Likens 1989; Lindenmayer et al. 2012), but funding and other challenges hinder such efforts (Schradin and Hayes 2017), and this is even truer when one considers long‐term research of ecological assemblages. Additionally, the intellectual benefits also increase when multiple interacting species are studied simultaneously (Brown 1998; Dickman et al. 2010; Greenville et al. 2016; Jiang et al. 2015; Krebs et al. 2019; Meserve et al. 2003; O'Connell and Hallett 2019).

Arid lands have played a prominent role in the development of ecological theory. To some extent, this likely is due to the more sparse vegetative communities found in such habitats, which makes individual organisms easier to distinguish and study (e.g., Ignace et al. 2018), and facilitates effective field experimentation such as biotic exclosures (e.g., relative to densely canopied forests and shrublands). Additionally, deserts and semi‐deserts are widespread and geographically disjunct; they generally support distinct evolutionary lineages that demonstrate variable levels of ecological convergence (Kelt 2011; Mares 1979). They also tend to be attractive to human intervention, leading to conservation issues when urban regions overlap areas of high biological diversity (Ezcurra 2006). Also, arid lands are expanding due both to climate change and more proximate influences such as overgrazing, removal of fuels, over‐cultivation, and human urban expansion (Ezcurra 2006; Ward 2016).

We have been studying an ecological community in semiarid north‐central Chile since 1989, when we established 16 replicate study plots to experimentally assess the relative influence of predation and inter‐specific competition on small mammals and, indirectly, on plants (Armas et al. 2016; Gutiérrez et al. 2010; Kelt et al. 2013; Meserve et al. 2003, 2011). In the present contribution, we used data solely from four control plots to which all species have had free access since the inception of this study. Of particular relevance is that the small mammal community is diverse, and with few exceptions, we have monitored the full community with four consecutive nights of live trapping monthly since April 1989 (details in Methods). Here, we use data through December 2020 (e.g., > 30 years) to characterize population dynamics of small mammals at our study site and to evaluate drivers of demographic trends in a suite of ecologically and phylogenetically distinct small mammals. We apply modern capture–mark–recapture (CMR) models to more than 30 years of monthly CMR data to address the following overarching questions: (i) How do population sizes and associated demographic parameters in our focal species vary seasonally and over time? And (ii) Are there commonalities across species in seasonal and annual variation in demographic parameters and population dynamics? More specifically, for each species, we estimate demographic parameters and population size, quantify seasonal variation (using both reproductive and climatic seasons), and assess how these population parameters are affected by patterns of annual rainfall. We also tested for any sex‐specific differences in demographic rates. To our knowledge, this comprises a unique attempt to characterize fundamental population parameters using CMR modeling of such extensive sampling (30 years of monthly capture–recapture data) for all species in a small assemblage.

## 2 Methods

### 2.1 Study Area and Focal Species

**FIGURE 1:**
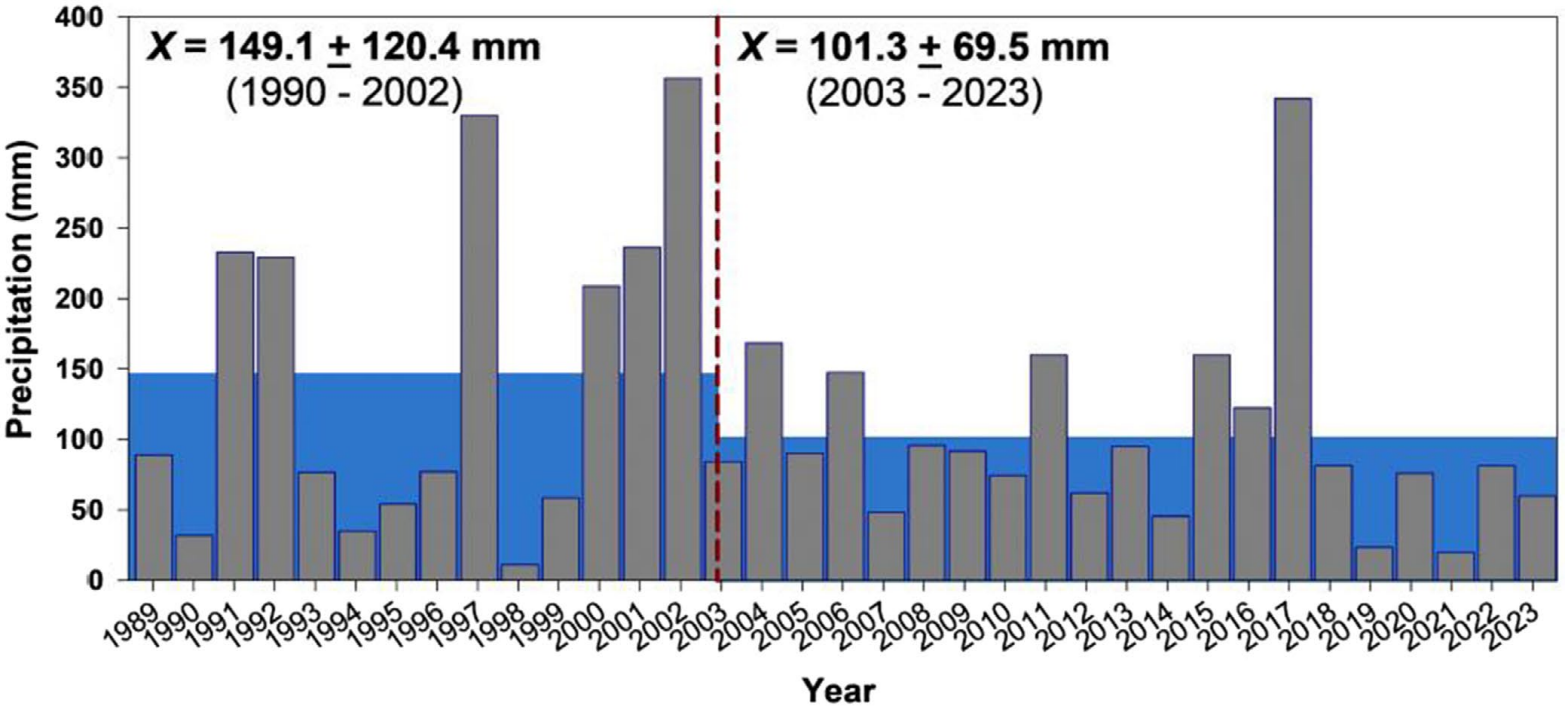
Rainfall at Fray Jorge, 1989–2023. Blue highlighting illustrates the mean annual rainfall before and after 2002–2003.

Bosque Fray Jorge National Park (30°38′ S, 71°40′ W; Fray Jorge hereafter) is located on the coast of north‐central Chile, near the northern end of the Mediterranean Region of Chile (ca. 380 km N Santiago), about 150 km south of the hyper‐arid Atacama Desert, and within a region known as the Norte Chico. Declared a national park in 1941 and a UNESCO World Biosphere Reserve in 1977, the ca. 9000 ha area has been managed by Chile's Corporación Nacional Forestal (CONAF) and has been largely free of grazing or other disturbances for half a century (Squeo et al. 2004). The climate is semiarid Mediterranean, with about 90% of annual rainfall (ca. 120 mm, 1989–2023) falling in the Austral winter (May–September); the Austral summer months (December–February) are warm and dry. The park is dominated by semiarid thorn‐scrub vegetation 2–3 m in height, although coastal hills support fog forests (Squeo et al. 2016). The thorn scrub includes spiny drought‐deciduous and evergreen shrubs and both ephemeral and perennial understory vegetation on a largely sandy substrate (Gutiérrez et al. 1993; Muñoz 1985; Muñoz and Pisano 1947). Fray Jorge is spatially heterogeneous, and data suggest that some species are more abundant in non‐thorn‐scrub habitats. Two such habitats warrant attention; these are the fog forests, mentioned above, and “aguada” habitat at the bottom of the valley. Aguadas are also more mesic than the thorn scrub, as this is where episodic rain runoff drains the valley (Milstead et al. 2007). Since the beginning of this study (1989) there have been seven El Niño/high rainfall events: 1991–1992 (233, 229 mm), 1997 (330 mm), 2000–2002 (209, 236, & 356 mm), 2004 (168 mm), 2006 (147 mm), 2011 (160 mm), 2014–2016 (160, 122, & 342 mm); intervening years have been dry (mean 64 mm, range 11–96 mm). Rainfall patterns appear to have shifted since about 2003, with significant declines in both the mean annual rainfall and variance (Figure 1).

**FIGURE 2:**
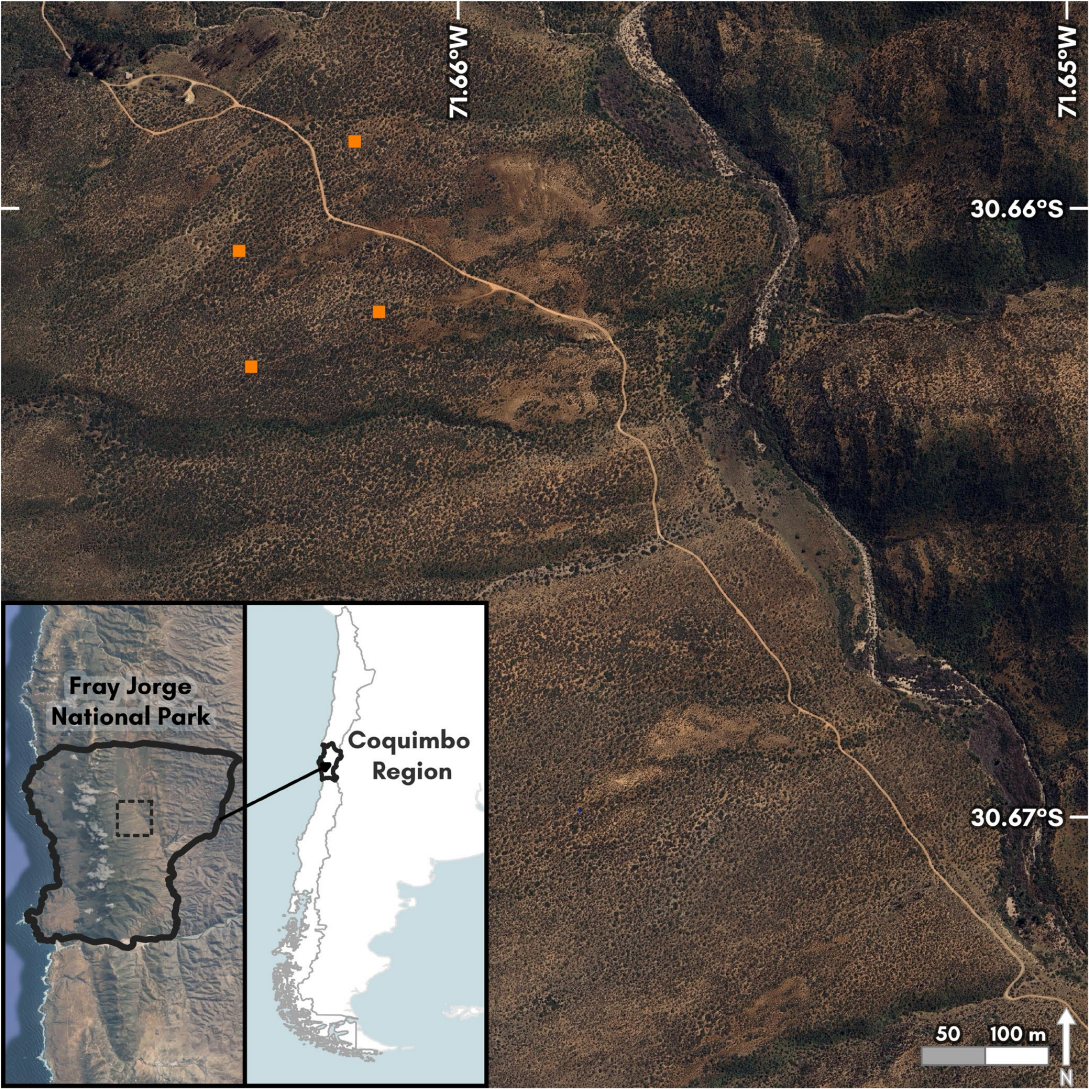
Location of four study plots in the Quebrada de las Vacas, Bosque Fray Jorge National Park. Fog forests occur at the top of the Altos de Talinay, to the left in this image, while aguada habitat occurs along the drainage of the Quebrada, descending north to south at the right of the image. Plot symbols do not indicate plot size. Cartography by Michele M. Tobias, UC Davis DataLab. Background data: OpenStreetMap, Natural Earth Data, and Google Maps.

In 1989 we established a series of permanent live‐trapping grids in a valley (Quebrada de las Vacas, ca. 240 m elev.) located immediately inland from a coastal range (Altos de Talinay, ca. 600 m elevation). Whereas the original study design included four replicates of four treatments, including exclusion of predators (with fencing and netting), a key rodent, the Common Degu ( *Octodon degus* ), or both, we limit our efforts here to four control plots that allowed free access by all species (Figure 2). Parenthetically, we note that movements between plots do occur, but these are relatively uncommon, such that predator‐exclusion plots are unlikely to serve as “refuges” for animals on control plots reported on here. Plots are 75 × 75 m (=0.56 ha) and treatments were randomly arranged within comparable habitats (see Kelt et al. 2013 for details). Since April 1989, we have surveyed all plots for four nights and 3 days each month, using 5 × 5 grids (15‐m spacing, two 300 × 100 × 100 mm aluminum “Sherman type” live traps per station), with rare exceptions due to excessive rains. Traps are baited with rolled oats and set in late afternoon, then checked early in the morning; they are then reset and rechecked at midday to account for diurnal species (e.g., *O. degus* ). Monthly effort, therefore, is 400 trap‐nights plus 300 trap‐days per plot. Captured animals are gently removed from traps, identified to species, sex determined, and weight recorded (using a spring scale) before being released at the point of capture. All animals are permanently identified with either uniquely numbered ear tags or (for animals too small to support ear tags) uniquely numbered leg bands; the latter are removed and replaced with ear tags when animals grow large enough to support the latter. Between March 1989 and December 2019, we monitored small mammals for four nights and 3 days monthly over 370 continuous months (minus one night in June 1992 due to excessive rain), resulting in 517,600 trap‐nights/days of effort on four control plots; efforts were paused in 2020 in response to the COVID‐19 pandemic, but all data used herein predate the pandemic. All field activities followed guidelines recommended by the American Society of Mammalogists (Sikes and Animal Care and Use Committee of the American Society of Mammalogists 2016) and were approved by the University of California Davis Animal Care and Use Committee as well as the CONAF and Servicio Agrícola y Ganadero in Chile.

The small mammal fauna of Fray Jorge (Fulk 1975; Meserve 1981a, 1981b; Meserve and Le Boulengé 1987; Schamberger and Fulk 1974) includes the Elegant Fat‐tailed Opossum ( *Thylamys elegans* , ca. 25–35 g), several caviomorph rodents such as the diurnal and highly social Common Degu (ca. 120–180 g), the Moon‐toothed Degu ( *O. lunatus* , ca. 160–200 g), and Bennett's Chinchilla Rat ( *Abrocoma bennettii* , ca. 150–250 g), and several sigmodontine rodents, including the Olive Field Mouse (*Abrothrix olivacea*, ca. 20–40 g), the Long‐haired Field Mouse ( *Abrothrix longipilis* , ca. 30–50 g), Darwin's Pericote ( *Phyllotis darwini* , 40–60 g), the Long‐tailed Pygmy Rice Rat ( *Oligoryzomys longicaudatus* , 20–35 g), and the rare Chilean Long‐clawed Mouse ( *Chelemys macronyx* , ca. 20–55 g). The fully fossorial Coruro ( *Spalacopus cyanus* , ca. 70–120 g), also a caviomorph, is common at Fray Jorge but is rarely captured in our traps. Caviomorph and sigmodontine rodents generally differ in lifespan and reproductive rates (Hayssen et al. 1993; Rowlands and Weir 1974).

Our sampling efforts yielded 75,994 captures of 28,355 individual small mammals, representing nine species. Two species were sufficiently uncommon ( *C. macronyx* and *O. lunatus* , 13 and 100 captures of 6 and 55 individuals, respectively) that further analyses were not pursued. Seven other species (Table 2) comprise the bulk of our data, with a combined total of 75,881 captures of 28,294 individuals. These include the didelphid marsupial, *T. elegans* , and six rodents, the caviomorphs, *A. bennettii* and *O. degus* , and the sigmodontines, *A. olivacea* , *A. longipilis* , *O. longicaudatus* , and *P. darwini* .

We classify small mammals as either core or opportunistic elements of the thorn‐scrub habitat. Core species are present in nearly all monthly surveys and therefore comprise the majority of our captures. Opportunistic species may be absent for many months, and we believe that they recede to more mesic habitats such as fog forest and the aguadas; when their populations increase, they appear to spill over to our thorn‐scrub sites. Core species include *O. degus* , *P. darwini* , and *A. olivacea* . *T. elegans* is present in almost all months and may be under‐sampled due to its semi‐arboreal nature; as such, we refer to this species as a quasi‐core element of the small mammal fauna. Opportunistic species include all remaining species. *A. longipilis* appears to favor the fog forests, whereas *O. lunatus* appears to be more common in aguada habitat. *A. bennettii* and *O. longicaudatus* are found in both of these habitats but may favor aguadas, although further research is needed to confirm this. *C. macronyx* is found almost exclusively in the fog forests at Fray Jorge, which support a limited and disjunct population (Teta et al. 2015).

### 2.2 Data Analysis and Modeling

We used the superpopulation CMR modeling framework (Schwarz and Arnason 1996; Williams et al. 2002) to estimate population size and demographic parameters that cause changes in population size over time. The superpopulation model is a reparameterization of the Jolly‐Seber CMR model (Jolly 1965; Seber 1965). This modeling approach envisions that there exists a “superpopulation” (N*), defined as the sum of all individuals that would ever enter the population, and each member of the superpopulation enters the population at time *i* with probability *b*_*i*_. The net number of new individuals entering the population at occasion *i* is estimated as *E*[*B*_*i*_] = *Nb*_*i*_. Assuming that *B*_0_ represents the number of individuals in the population just before the first sample, the superpopulation size is given by

$$N^{*}=\sum_{i=0}^{k-1}{\hat{B}}_{i},$$

where *k* is the final sampling occasion. In addition to N*, this modeling approach estimates *b*_*i*_ (often referred to as probability of entry (PENT), the probability that a member of a superpopulation enters the study population between occasion *i* and *i* + 1), *φ*_*i*_ (apparent survival probability, the probability that a an individual that is alive and in the sampled population at occasion *i* is alive and in the sampled population at occasion *i* + 1), and *p*_*i*_ (capture probability or detectability, trappability; the probability that an individual is captured at occasion *i* given that it is alive and present in the sampled area). Once *b*_*i*_, *φ*_*i*_, and *p*_*i*_ are estimated, and recognizing that it is not possible to separate recruitment from in situ reproduction and immigration, the number of net recruits entering the population between occasions 𝑖 and 𝑖 + 1 (*B*_*i*_; hereafter, “recruits”), and time‐specific population size *N*_*i*_ is estimated as derived parameters as Williams et al. (2002):

$${\hat{B}}_{i}={\hat{N}}^{*}{\hat{b}}_{i}\;\text{and}$$

$${\hat{N}}_{1}={\hat{B}}_{0}$$

$${\hat{N}}_{2}={\hat{N}}_{1}\varphi_{1}+{\hat{B}}_{1}\;\text{and generally}$$

$${\hat{N}}_{i+1}={\hat{N}}_{i}\varphi_{i}+{\hat{B}}_{i}$$

**FIGURE A1:**
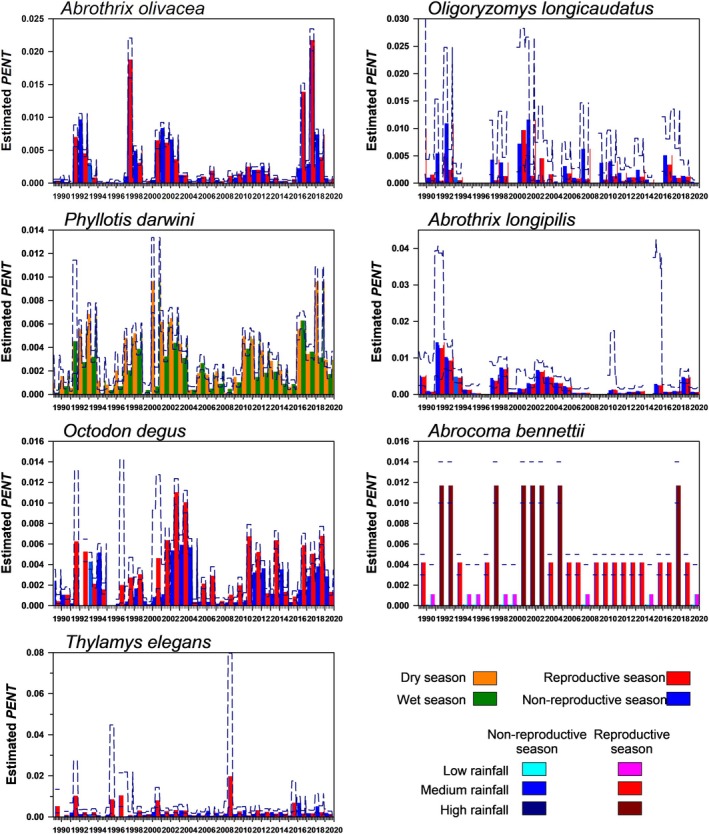
Probability of entry (*PENT*) for core (left) and opportunistic (right) species at Bosque Fray Jorge National Park.

All CMR analyses were conducted in Program MARK (White and Burnham 1999) using the RMark package (Laake 2013) for the R computing environment (R Core Team 2024). Because of large sample sizes for most species (360 sampling occasions, 599–25,083 captures of 511–9775 unique individuals; Table 2), fitting CMR models was computationally demanding. To reduce the number of models, we adopted a sequential approach to model fitting (Rolland et al. 2021). First, for each species, we conducted preliminary analyses to determine the best general structure(s) for capture probability (trappability, *p*; Appendix Table A1). For these analyses, we allowed *p* to be affected by sex, season, year, annual rainfall (dry or wet years), additive and interactive effects of the aforementioned variables, and when data permitted, time (see Table 1 for covariates); N* was modeled as constant or sex‐specific; model structures for *PENT* and *φ* were allowed to be fairly general (this varied across species, depending on data availability). For subsequent analyses, the model structure for *p* was fixed to the top (or top two) model structures determined from the previous step; we allowed N* to be constant or sex‐specific, whereas *PENT* and *φ* were allowed to vary by sex, climatic season (wet/dry), reproductive season, year, and both binary and trinary metrics of annual rainfall (Table 1), as well as both additive and interactive effects of these variables representing specific hypotheses. Our binary metric of rainfall classifies years as dry or wet if annual rainfall is below or above the long‐term mean of 125 mm, respectively; the trinary metric attempts to separate central tendency by defining low, medium, and high rainfall years using the first, second plus third, and fourth quartiles in rainfall, respectively. Because reproductive activity at this semiarid site is strongly influenced by primary productivity (e.g., Meserve and Le Boulengé 1987; Previtali et al. 2009), we applied a common definition of these seasons for all species. This is supported by a simple metric of activity (proportion of females reproductively active) which suggests that for most species, more than 20% of the female population is reproductively active from September through November; for two core species ( *A. olivacea* and *P. darwini* ) there is support for reproductive activity into December as well, but these species also show signs of limited reproductive activity in every month of the year. For one species ( *O. longicaudatus* ) data were insufficient to support these models; thus, we investigated and fitted simpler model structures, which we detail in Results. Individuals of some species were not captured in some sampling occasions; we fixed *p* = 0 for sampling occasions when individuals of a particular species were not captured. For species with a small sample size (e.g., *A. bennettii* ; 599 captures of 511 individuals), we were compelled to fit simpler models because of data limitations. Because the interpretation of *PENT* is not intuitive, we present results for this parameter, but we do not discuss this in any depth (see Appendix Figure A1). Models were run on the University of California Davis Farm Cluster, a Linux‐based supercomputer cluster providing 512 GB of RAM.

**TABLE 1:** Table of model parameters.

Covariate	Parameterization
repro_season	Breeding season: June–November Non‐breeding season: December–May
rain_season	Rainy season: May–October Dry season: November–April
Rainfall	Annual, measured onsite: mm
Rain_LMH	Our trinary metric of annual rainfall (low/medium/high); **Low (bottom quartile)**: 1990, 1994, 1995, 1998, 1999, 2007, 2014, 2019; **Med (2nd and 3rd quartiles)**: 1989, 1993, 1996, 2003, 2005, 2006, 2008, 2009, 2010, 2011, 2012, 2013, 2015, 2016, 2018; **High (upper quartile)**: 1991, 1992, 1997, 2000, 2001, 2002, 2004, 2017
Wet_Dry	Our binary metric of annual rainfall (above/below long‐term mean of ca. 125 mm); **Dry (< 125 mm)**: 1989, 1990, 1993, 1994, 1995, 1996, 1998, 1999, 2003, 2005, 2007, 2008, 2009, 2010, 2012, 2013, 2014, 2016, 2018, 2019; **Wet (> 125 mm)**: 1991, 1992, 1997, 2000, 2001, 2002, 2004, 2006, 2011, 2015, 2017

We used an information‐theoretic approach for model selection, with Akaike's information criterion corrected for small sample size (AIC_c_) as a measure of model parsimony (Burnham and Anderson 2002; Williams et al. 2002). Covariate effects were assessed by comparing AIC_c_ among models with and without covariates. Models with a difference in AICc (ΔAICc) ≤ 2 were considered to be equally plausible. Estimates of the number of recruits are based on the top model, but unless otherwise indicated, we report all other parameters of interest as means of model‐averaged point estimates and either ranges (text) or 95% confidence limits (figures).

## 3 Results

### 3.1 Demographic Parameters—Core Species

*A. olivacea* is a sigmodontine rodent and the smallest core species at Fray Jorge. Reflecting its size, this species responds to favorable conditions relatively rapidly (Meserve, Kelt, et al. 2016; Meserve et al. 2003). During the study period, we recorded 24,135 captures of 9773 individuals (3318 females, 6455 males). Model‐averaging proceeded well for this species, and we report model‐averaged parameters here, although the top two models performed substantially better at describing patterns for this species than subsequent models (∆AICc for the 3rd model = 6.28, *w*_*i*_ for the 1st two models = 0.98; Table 3).

**FIGURE 3:**
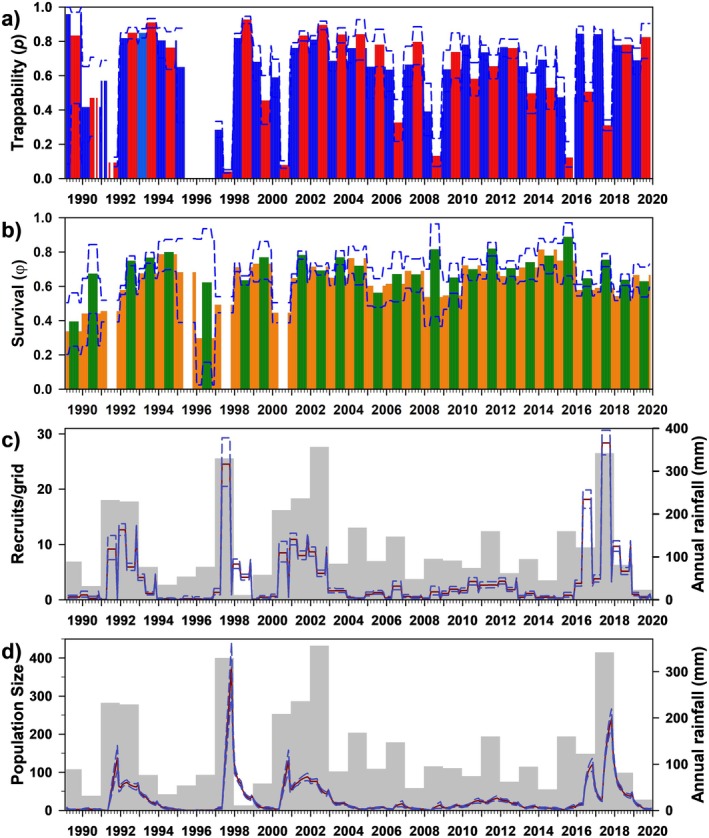
Estimates of trappability (a: females only; *p*), survival (b: *φ*), number of recruits (c), and population size (d) for *Abrothrix olivacea* at Bosque Fray Jorge National Park, Chile. For *p* and *φ*, vertical bars represent the model‐averaged point estimates, whereas dashed lines represent upper and lower 95% confidence limits; gaps represent sessions when parameters were not estimable due to insufficient data. Bar colors correspond to model parameterization (Table 3); red/blue = breeding/non‐breeding seasons, green/orange = rainy/dry seasons. For the bottom two panels, light gray bars represent annual rainfall, whereas lines provide the mean (red) and 95% CI (blue) of the estimates of the number of recruits and model‐averaged population size.

Trappability (*p*) was best modeled as a function of year interacting with reproductive season, with an additive effect of sex (Figure 3a). Using the best model, trappability varied greatly (mean 0.633, range 0.035–0.959), with little variation across seasons (breeding, 0.590, 0.035–0.928; non‐breeding, 0.674, 0.254–0.959) and sexes (females, 0.647, 0.041–0.959; males, 0.618, 0.035–0.953). Monthly survival (*φ*) was best modeled as a function of year interacting with rainfall seasons (Figure 3b), and also remained high but temporally variable (0.660, 0.298–0.889; hence, annual survival = 0.660^12^ ≈ 0.007), with slightly higher survival in dry seasons (0.706, 0.396–0.889) than in wet seasons (0.620, 0.298–0.815).

**FIGURE A2:**
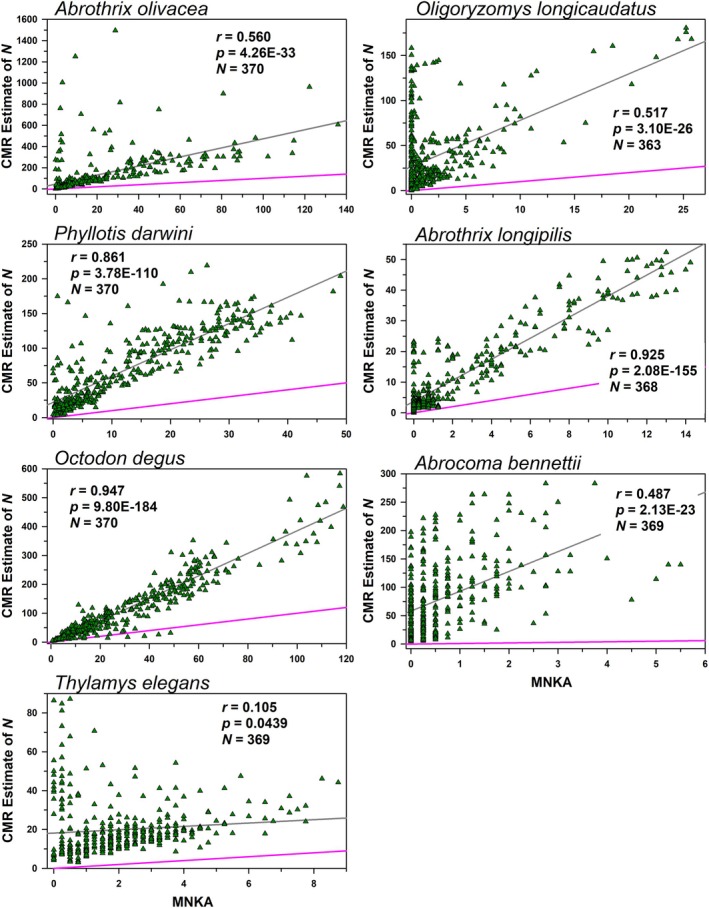
Correlation of population estimates obtained by traditional Minimum Number Known Alive (MNKA) and by the superpopulation capture‐mark‐recapture approach employed in this manuscript. The pink line is a line of equivalency; as CMR adjusts minimum capture estimates by trappability, all values should lie above this line; of interest is the extent to which CMR estimates deviate from the line, and the consistency with which they do. The latter is quantified here by the best linear fit (dark gray line) and results of a Pearson correlation between the two sets of variables.

The estimated number of recruits/grid varied greatly (8.807, 0.405–70.874), and not surprisingly, given the influence of rainfall in both survival and *PENT* (Table 3), was strongly associated with rainfall (Figure 3c). Finally, model‐averaged estimates of population size varied tremendously in this species (30.215/grid, 0.240–374.010), with an estimated sex ratio (M/F) of 2.03. General patterns in estimated population size deviated somewhat from the minimum number known alive (MNKA) (*R* ^2^_adj_ = 0.32), and CMR estimates are, on average, 15.33 times higher than MNKA (range 1.68–338.20; Appendix Figure A2).

*P. darwini* is larger than *A. olivacea* but also a sigmodontine rodent, and so these species share more rapid reproductive potential relative to caviomorphs at this site. During the study period, we recorded 17,940 captures of 8316 individuals (3107 females, 5209 males). Model‐averaging proceeded smoothly for this species, although the top model was substantially better than any other model (∆AICc = 48.27, *w*_*i*_ ≈ 1.0; Table 3).

**FIGURE 4:**
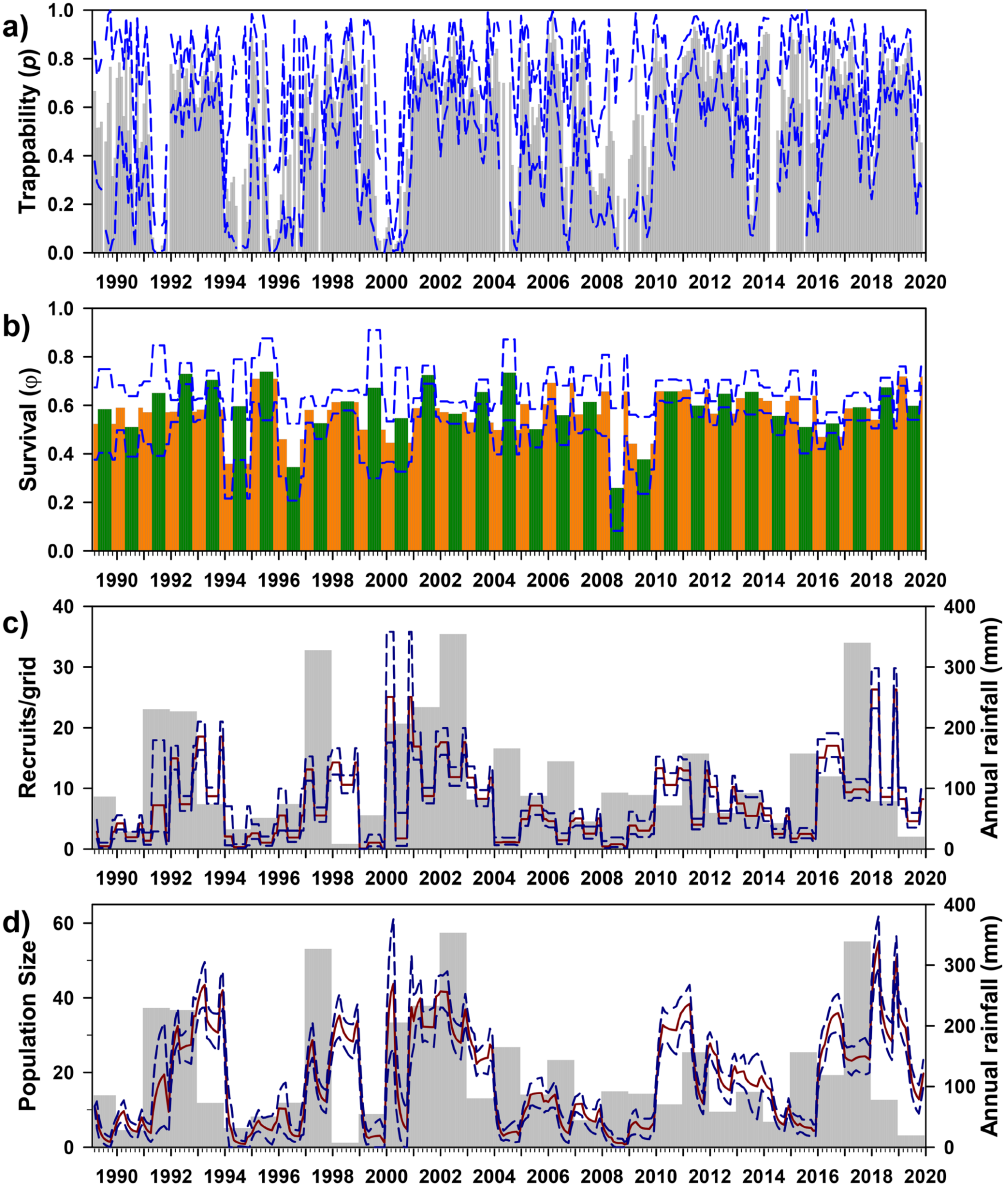
Estimates of trappability (a: *p*), survival (b: *φ*), number of recruits (c), and population size (d) for *Phyllotis darwini* at Bosque Fray Jorge National Park, Chile. Panels as in Figure 3. Bar colors correspond to model parameterization (Table 3); gray = full time‐dependence, green/orange = rainy/dry seasons.

Trappability (*p*) varied greatly (0.597, 0.011–0.972) and was best modeled as a simple function of time, with only trivial differences across seasons or sexes (Figure 4a). Monthly survival (*φ*) was best modeled as an interaction of year and rainfall seasonality (e.g., wet/dry seasons; Figure 4b). Survival was moderately high throughout most of the study period (0.584, 0.265–0.742; hence, annual survival = 0.584^12^ ≈ 0.002), and differed only slightly between wet (0.592, 0.265–0.742) and dry seasons (0.576, 0.363–0.722).

The estimated number of recruits/grid varied temporally (7.197, 0.077–26.286), also seemingly strongly influenced by annual rainfall, albeit with a few anomalies (e.g., 1993, 2018, 2019; see Figure 4c). In addition, as with *A. olivacea* , model‐averaged estimates of population size generally varied in parallel to the number of recruits, including demographic expansion in relatively arid years that follow particularly wet years (Figure 4d). Further reflecting *A. olivacea* , population size varied greatly (17.870/grid, 0.708–54.796), with a male‐biased sex ratio (M/F = 1.70). Finally, CMR estimates of *N* tracked MNKA estimates modestly well (*R* ^2^_adj_ = 0.74), although the former were, on average, 10.69 times higher (range, 2.04–233.22; Appendix Figure A2).

The third core rodent species is *O. degus* , a relatively large and diurnal caviomorph endemic to central Chile. Our data represent 25,178 captures of 6099 individuals (2993 females, 3106 males). Model‐averaging proceeded smoothly for this species, producing two competitive models that differ solely in parameterization for superpopulation size (Table 3).

**FIGURE 5:**
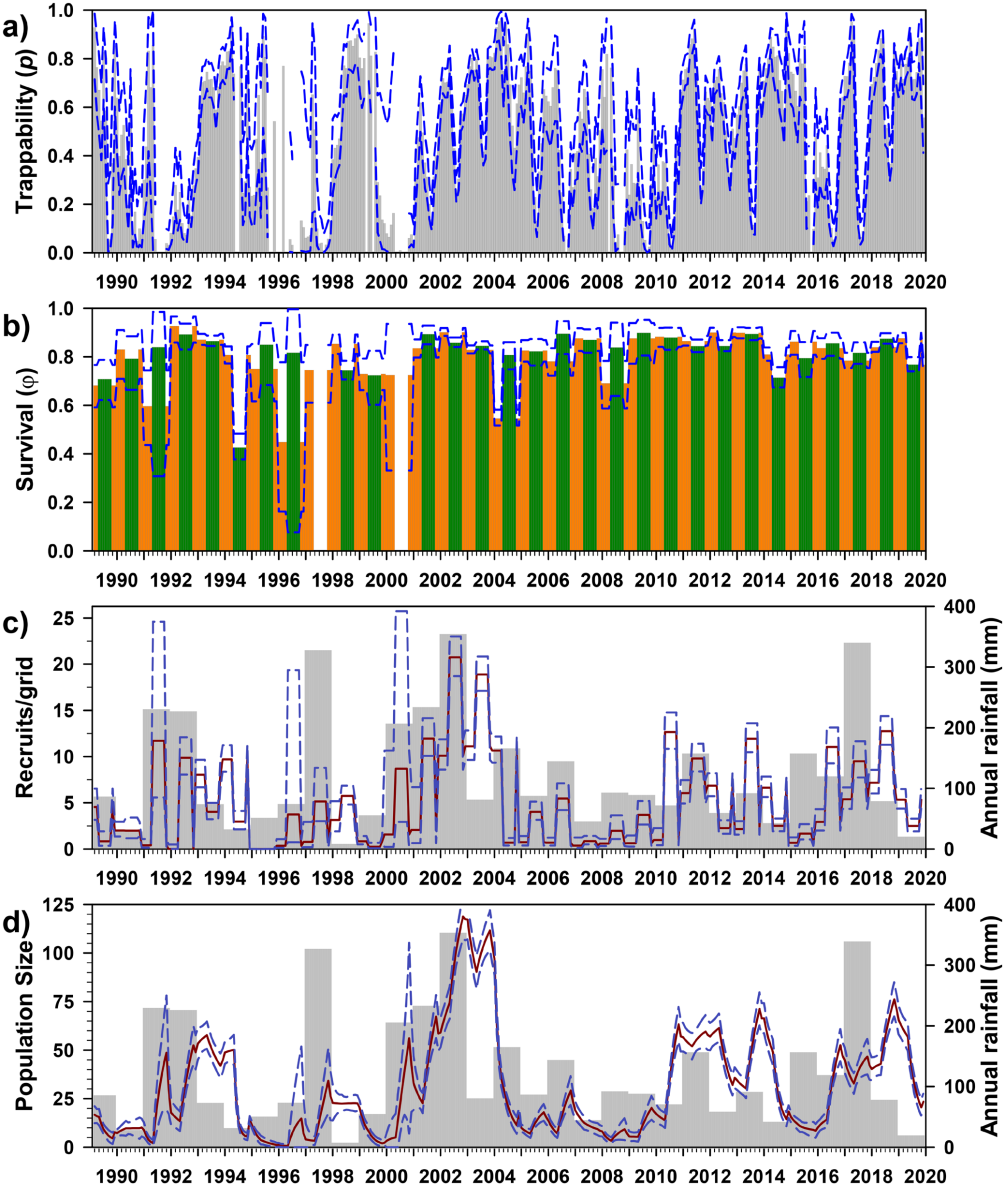
Estimates of trappability (a: *p*), survival (b: *φ*), number of recruits (c), and population size (d) for *Octodon degus* at Bosque Fray Jorge National Park, Chile. Panels as in Figure 3. Bar colors correspond to model parameterization (Table 3); gray = full time‐dependence, green/orange = rainy/dry seasons.

Trappability (*p*; Figure 5a) was quite variable, and as with *P. darwini* , was best modeled solely as a function of time (0.502, 0.011–0.965). Monthly survival (*φ*) was best modeled as an interaction between year and rainfall season (wet/dry; Figure 5b), and generally remained high (0.809, 0.430–0.930; hence, annual survival = 0.809^12^ ≈ 0.079) throughout most of the study period; seasons were quite similar overall (dry, 0.800, 0.452–0.930; wet, 0.819, 0.430–0.902).

Estimates of the number of recruits/grid varied greatly over time (5.204, 0–20.731), evidently responding to rainfall but with a notable lag (Figure 5c). Model‐averaged population size varied greatly over time (30.829/grid, 0.678–118.759; Figure 5d) and parallel to the number of recruits. The estimated sex ratio (M/F) was 1.04. Finally, estimated population size varied closely with MNKA (*R* ^2^_adj_ = 0.90), but on average was 3.78 times higher (range 0.59–95.45; Appendix Figure A2).

The final core (or quasi‐core) species at Fray Jorge is the Elegant Fat‐tailed Mouse Opossum, *T. elegans* . We recorded 2437 captures of 1179 individual mouse opossums (467 female, 712 male). Model‐averaging proceeded well for this species, but there was one clear top model (*w*_*i*_ ≈ 0.76) and one marginally subordinate model (∆AICc ≈ 2.30, *w*_*i*_ ≈ 0.24) that differs solely in parameterization for *p*.

**FIGURE 6:**
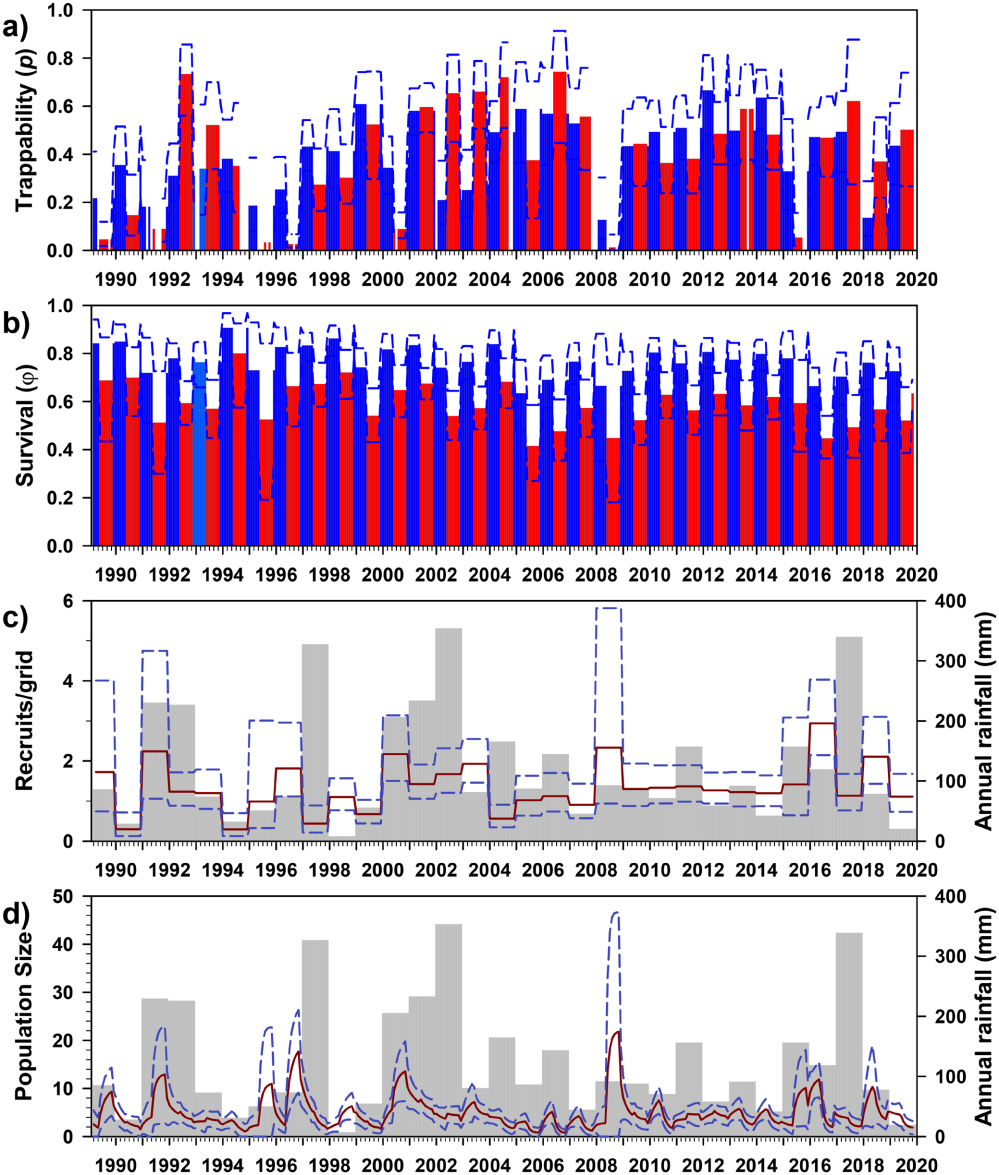
Estimates for trappability (a: *p*), survival (b: *φ*), number of recruits (c), and population size (d) for *Thylamys elegans* at Bosque Fray Jorge National Park, Chile. Panels as in Figure 3. Bar colors correspond to model parameterization (Table 3); red/blue = reproductive/non‐reproductive seasons.

Trappability (*p*) varied greatly over time, and was best modeled either as an interaction between year and reproductive season, or as the same with an additive effect of sex (Figure 6a); resulting parameters differed only slightly by sex (effectively identical to three significant digits; 0.416, 0.015–0.745), and illustrate similarly trivial variation across reproductive seasons (breeding 0.420, non‐breeding 0.413). Survival (*φ*) was identical in males and females, and best modeled as a function of year plus breeding season (Figure 6b). Survival was relatively high (0.682, 0.420–0.909; hence, annual survival ≈ 0.01), and lower during the breeding season (0.591, 0.420–0.804) than in the non‐breeding season (0.774, 0.639–0.909).

As expected for a small, semi‐arboreal mouse opossum, estimates of the number of recruits/grid were low but variable over time (1.337, 0.297–2.939), and generally (but inconsistently) associated with rainfall (Figure 6c). Similarly, model‐averaged population size was relatively low throughout the study, but notably variable (4.978/grid, 0.748–21.795); even its response to rainfall (included in the best parameterization of *φ*) seemed variable (e.g., 2009). The sex ratio (M/F = 1.547) was male‐biased. In particular, demographic expansions in 1996 and 2008/09 seem poorly linked to previous rainfall. Finally, estimates of population size varied notably from MNKA estimates (*R* ^2^_adj_ = 0.008), likely due in part to small sample sizes, which permitted only simple model structures. Estimates of population size were, on average, 21.63 times higher than MNKA (range, 2.29–339.22; Appendix Figure A2).

### 3.2 Demographic Parameters—Opportunistic Species

**FIGURE 7:**
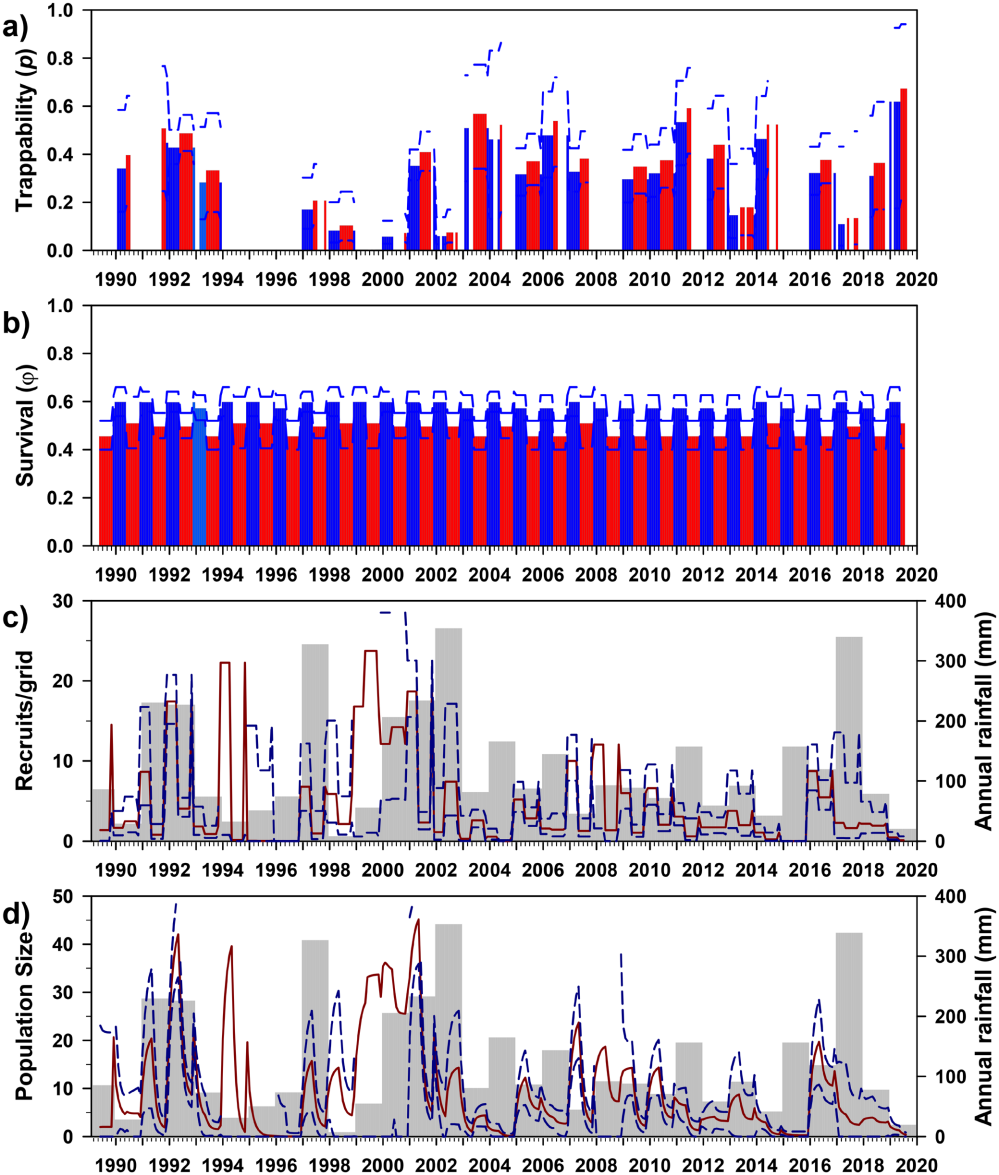
Estimated trappability (a: *p*), survival (b: *φ*), number of recruits (c), and population size (d) for *Oligoryzomys longicaudatus* at Bosque Fray Jorge National Park, Chile. Panels as in Figure 3. Bar colors correspond to model parameterization (Table 3); red/blue = reproductive/non‐reproductive seasons.

Reflecting the irruptive demography of the Long‐tailed Pygmy Rice Rat, *O. longicaudatus* (Meserve et al. 1991; Meserve et al. 1995; Murúa et al. 1986), we recorded only 2596 captures of 1782 individuals (575 females, 1207 males). Our “best model” could not estimate several parameters due to insufficient data, so we calculated model‐averaged parameter estimates based on the subsequent five models (all within ∆AICc < 5.5). Whereas the superpopulation was best parameterized by sex, sex had no effect on other parameters (Table 3). Although trappability (*p*; Figure 7a) was best modeled as an additive function of year and reproductive season (0.338, 0.005–0.675), it did not differ substantially across seasons (breeding, 0.359, 0.005–0.675; non‐breeding, 0.319, 0.060–0.620). Monthly survival (*φ*; Figure 7b) was best modeled as a function of reproductive seasons or reproductive season and a trinary measure of annual rainfall (either additive or interactive). Mean survival was 0.536 (range, 0.460–0.602, suggesting annual survival ≈ 0.0006), was lower in the breeding season than the non‐breeding season (0.483, 0.460–0.514 vs. 0.589, 0.576–0.602, respectively), and was slightly lower in years of medium rainfall (0.516, 0.460–0.576) than in high (0.550, 0.500–0.601) and low rainfall (0.559, 0.514–0.602).

The estimated number of recruits averaged 3.867 individuals/grid (range, 0.005–33.283; Figure 7c). As expected for an opportunistic species for which we believe xeric thorn scrub to be secondary habitat, model‐averaged population size was relatively low throughout the study, but also notably variable (9.169/grid, 0.004–45.166; Figure 7d). The estimated sex ratio (M:F) was male‐biased (2.149), possibly reflecting differential dispersal from natal (mesic) territories by male individuals. Finally, estimates of population size did not vary closely with MNKA (*R* ^2^_adj_ = 0.27), likely reflecting the interpolation of estimates provided by the latter; MNKA estimates of zero animals occurred in 148 of 366 estimated months, and during these “zero months,” CMR estimated an average of 31.002 animals (range, 0.018–158.333). Across all remaining months (e.g., MNKA > 0), CMR estimates were, on average, 38.931 times higher than MNKA estimates (range, 2.667–537.507; Appendix Figure A2).

*A. longipilis* is much larger than its core species congener ( *A. olivacea* ), but like *O. longicaudatus* it appears to favor more mesic habitat (Milstead et al. 2007), and so “spills over” to our thorn‐scrub plots during times of demographic expansion. We recorded 2996 captures of 634 individuals (227 females, 407 males) of this species during the study period. Model‐averaging proceeded well, yielding four “competitive” models (∆AICc < 2; Table 3), and four more with ∆AICc < 4; the weight of evidence in favor of the top model was modest (*w*_*i*_ = 0.29), and the following three models each added 0.11–0.15 such that the Akaike weights for the first four models summed to 0.67; all subsequent models contributed minimally (all *w*_*i*_ < 0.10). The top four models varied in parameterization for *φ* (year + reproductive season vs. year + rainfall season) and *PENT* (year + either reproductive season, rainfall season, or a binary metric of annual rainfall; Table 3).

**FIGURE 8:**
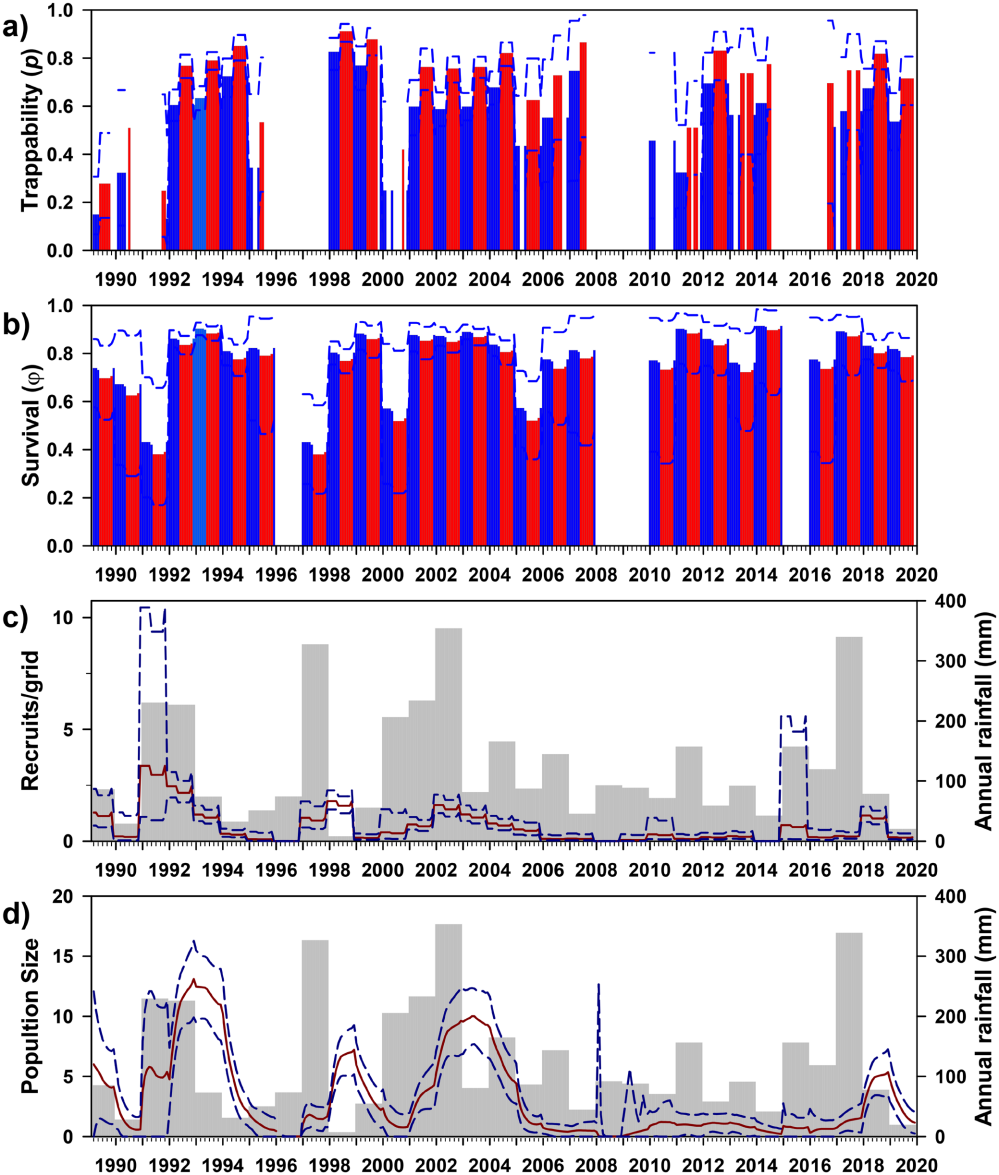
Estimated trappability (a: *p*), survival (b: *φ*), number of recruits (c), and population size (d) for *Abrothrix longipilis* at Bosque Fray Jorge National Park, Chile. Panels as in Figure 3. Bar colors correspond to model parameterization (Table 3); red/blue = reproductive/non‐reproductive seasons.

Trappability (*p*) was parameterized in all leading models by an additive effect of year plus reproductive season and was fixed at zero or unity in 127 (of 370) sessions when no individuals were captured (Figure 8a). Trappability generally was fairly high but varied substantially (0.649, 0.134–0.912) and was somewhat greater during the breeding season (0.732, 0.251–0.912) than during the non‐breeding season (0.574, 0.134–0.828). As noted above, monthly survival (*φ*) was best modeled as an additive function of year plus either reproductive season or rainfall season (Figure 8b). Survival generally was moderately high (0.768, 0.384–0.918; hence, annual survival = 0.768^12^ ≈ 0.042), was slightly lower in the breeding season (0.752, 0.384–3.904 vs. 0.784, 0.425–0.918), and slightly lower in wet years relative to dry years (0.756, 0.384–0.914; 0.781, 0.395–0.918).

The estimated number of recruits generally was low (0.623/grid, ≈0–3.365), but clearly associated with rainfall (Figure 8c). Reflecting this, model‐averaged population sizes were low in most months, with sporadic episodes of higher numbers, invariably following rainy years (3.119/grid, ≈0–13.092). The estimated sex ratio (M/F) was 1.857. Finally, the general patterns in estimated *N* regress well on MNKA estimates (*R* ^2^_adj_ = 0.85), although CMR estimates were, on average, 6.790 times higher than those from MKNA (range, 1.386–66.009; Appendix Figure A2).

Finally, *A. bennettii* is a caviomorph rodent and one of the largest species at our study site. As noted above, this species appears to be more abundant in “aguada” habitat where more mesic vegetation occurs (Milstead et al. 2007). Nonetheless, it is not an uncommon species on our sampling plots, and we recorded 599 captures of 511 individuals (271 females, 240 males). Limited captures mandated that we apply simpler models than for other species, and the most efficacious efforts were restricted to modeling *p*, *φ*, and *PENT* as invariant or influenced by reproductive season, a tripartite metric of annual rainfall, and both additive and interactive combinations of the above. Model‐averaging failed to converge with all models, so we proceeded with only the top ten models (Σ*w*_*i*_ = 0.982); this yielded three “competitive” models (∆AICc < 2), and two more with ∆AICc < 4, although only the first three had Akaike weights above 0.10, and the weight of evidence supporting the top three models was 0.75 (Table 3). All parameters were calculated with model‐averaging, although limited sampling and relatively low recapture rates resulted in very low estimates of trappability; this may result in anomalously high estimates of both recruitment and population size, which should therefore be interpreted with caution.

**FIGURE 9:**
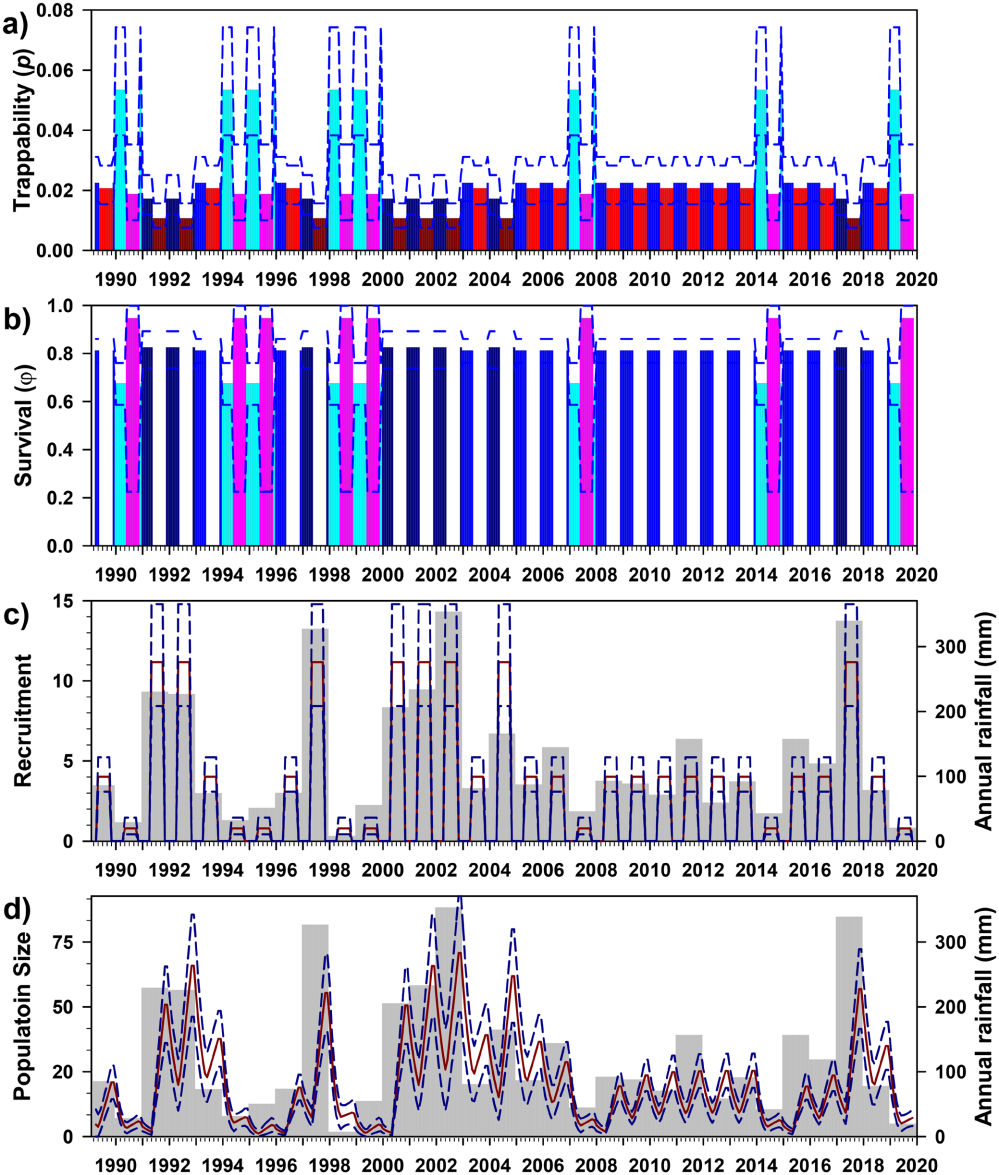
Estimated trappability (a: *p*), monthly survival (b: *φ*), number of recruits (c), and population size (d) for *Abrocoma bennettii* at Bosque Fray Jorge National Park, Chile. Panels as in Figure 3. Bar colors correspond to model parameterization (Table 3); shades of red (pink, red, dark red) = reproductive season * low, medium, and high rainfall years, respectively; shades of blue (cyan, blue, dark blue) = non‐reproductive season * low, medium, and high rainfall years, respectively.

Trappability was parameterized in all competitive models by an interaction between reproductive season and annual rainfall. Trappability (Figure 9a) was quite low (0.024, 0.011–0.054), was similar in breeding (0.018, 0.011–0.021) and non‐breeding seasons (0.029, 0.017–0.054), and declined from low (0.036, 0.019–0.054) to medium (0.022, 0.021–0.023) to high (0.014, 0.011–0.017) rainfall years. Overall, survival was moderately high (0.819, 0.680–0.950), suggesting annual survival = 0.819^12^ ≈ 0.09 (Figure 9b). Unlike *p*, survival was higher in the breeding seasons (0.950, no variation) than in the non‐breeding season (0.785, 0.680–0.829), but was slightly lower during low (0.817, 0.680–0.950) and medium (0.816, no variation) rainfall years than during high rainfall years (0.829, no variation).

The estimated number of recruits was low and strongly associated with annual rainfall (1.906/grid, ≈0–8.372; Figure 9c). Model‐averaged population size was relatively variable (19.886/grid, 1.153–70.771) and evidently associated with rainfall, although seemingly with a bit of a delay (Figure 9d). The estimated sex ratio (M/F) was even (0.98). Finally, the estimated population size reported here correlates poorly with MNKA estimates (*R* ^2^_adj_ = 0.24), although MNKA estimates of zero animals occurred in 130 of 369 estimated months; mean estimated N during these “zero months” was 12.209 animals (range, 1.153–49.344), whereas estimates across all remaining months were, on average 40.636 times higher than MNKA estimates (range, 3.563–221.589; Appendix Figure A2).

## 4 Discussion

Rainfall is considered to be the “master input” in many arid regions (Collins et al. 2014; Noy‐Meir 1973), and numerous studies from Chile (Holmgren et al. 2006; Jaksic 2001; Lima et al. 1999; Meserve et al. 2003; Meserve et al. 1999; Meserve et al. 2003), Australia (Bennison et al. 2018; Holmgren et al. 2006), and North America (e.g., Arizona, Brown and Ernest 2002; Ernest et al. 2000; Thibault et al. 2010; coastal California, Chaudhary et al. 2021; Polyakov et al. 2021; Rolland et al. 2021; Srivathsa et al. 2019; Tietje et al. 2018) generally supports the predominant role of rainfall in driving small mammal population and community dynamics. The past few decades have seen a proliferation of papers highlighting the role of ENSO events (and rainy years in general) on the biota of Mediterranean Chile (e.g., Holmgren et al. 2001; Jaksic 1998, 2001), and it is now generally recognized that biotic factors are subordinate to abiotic influences as structuring forces in this system, with the latter effectively “re‐setting the clock” every few years (Meserve, Kelt, et al. 2016; Meserve et al. 2003). Our current results are consistent with these observations, and reinforce our earlier finding that abiotic drivers are disproportionately important in this system (Meserve et al. 1999).

**FIGURE 10:**
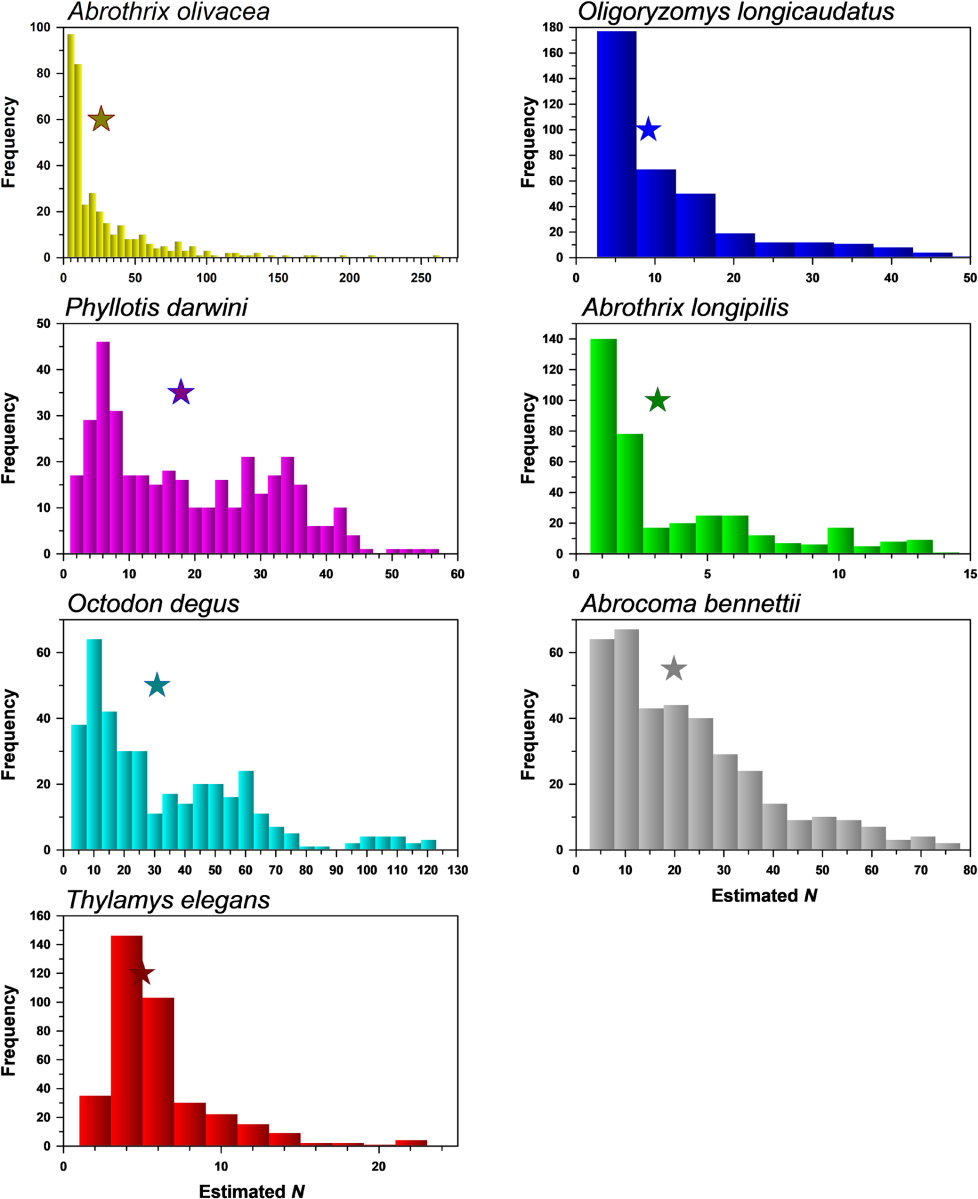
Frequency distributions for estimated *N* over 370 months for seven species at Fray Jorge. Four “core” species are given on the left; three “opportunistic” species are on the right. The star indicates mean *N* across all months.

The presence of rainfall‐related variables in nearly all well‐supported models for survival or entry probability (*PENT*) for most species included in the present study (Table 3), as well as the dominance of episodic years of high population growth following good rainfall years, and frequency distributions of estimated population size for all species with long tails reflecting uncommon, highly productive years (Figure 10), underscore the role of rainfall in driving the population dynamics of small mammals in this system, while offering deeper insight to the underlying mechanisms by which periodic mesic or wet periods lead to population increases. Reproductive season was another important covariate, affecting *PENT* of most species and survival of some species. The former reflects the combined influence of recruitment and immigration on the probability of new animals entering the population, but the fact that this is more influential than rainfall for three of four core species and at least one opportunistic species highlights that these species are well adapted to the episodic nature of moisture and bottom‐up drivers in this system. Whereas *PENT* was greater during the breeding season for most species, it was about equal in both seasons for *A. longipilis* , and substantially lower during the breeding season for *O. longicaudatus* . Interestingly, rainfall failed to enter any competitive models for *T. elegans* , and only the least supported of the “top models” of survival for *A. longipilis* ; for both species, reproductive season was more influential on survival. We do not believe that this should be interpreted to suggest that rainfall is not important; indeed, rainfall leads to the proliferation of food resources, which, in turn, allows for reproductive output. We note that didelphid marsupials generally tend to be distinctly seasonal breeders, and seasonality tends to increase with latitude (Voss and Jansa 2021). In the only comprehensive study on the demography of *T. elegans* , however, reproductive rates were distinctly seasonal, and the proportion of individuals in reproductive condition was positively related to annual rainfall (Lima, Stenseth, et al. 2001). The latter study was conducted at Reserva Nacional Las Chinchillas, near Aucó, located ca. 90 km ESE of Fray Jorge in the foothills of the Andes. Both Las Chinchillas and Fray Jorge have distinct rainy seasons, such that any direct or indirect effect of rainfall could present as a “seasonality” effect. Clearly, further work on the demography of this poorly known marsupial is warranted. More generally, for both *T. elegans* and *A. longipilis* , we believe that rainfall is “hidden” in these models due to the more immediate linkage of population size to both in situ recruitment and *ex situ* immigration.

Most of the species reported here have received limited scientific attention, at least in arid regions of their distribution. *O. longicaudatus* and *A. longipilis* , for example, are more stable elements of faunas in southern Chile, where their dynamics have been studied to a greater extent (e.g., Meserve et al. 1991; Murúa et al. 2003a; Murúa et al. 2003b; Murúa et al. 1986; Murúa et al. 1987). Quantitative efforts on small mammals in north‐central Chile have emphasized the dominant species, mostly *P. darwini* and *O. degus* , with lesser contributions on *A. olivacea* and *T. elegans* (Crespin and Lima 2006; Le Boulengé and Meserve 1984; Lima 2001; Lima and Jaksic 1998a, 1998b, 1999a, 1999b, 1999c; Lima, Julliard, et al. 2001; Lima et al. 1998; Lima et al. 2002; Lima et al. 2003; Lima, Stenseth, et al. 2001; Meserve and Le Boulengé 1987; Meserve et al. 1984; Meserve et al. 2001; Previtali et al. 2009). The present contribution is the first to characterize the demography of nearly the entire assemblage of small mammals (possibly the first such effort for all of South America), and to model their temporal dynamics in terms of dominant extrinsic drivers.

### 4.1 Core Species Share More Model Parameterization Than Do Opportunistic Species

The conceptual approach employed in our modeling efforts assumes a “superpopulation” for each species, which includes all individuals that entered our study populations throughout the duration of the study. The core species in this study—those species present in virtually every monthly survey and hence, presumably well adapted to this semiarid environment—all appear to be autochthonous elements of the Norte Chico fauna, with extensive histories in this arid region [ *O. degus* : (Cadenillas and D'Elía 2021; D'Elía et al. 2021; Opazo 2005); *P. darwini* : (Steppan and Ramirez 2015, based on Walker et al. 1984); *A. olivacea* : (Smith et al. 2001); *T. elegans* : (Boric‐Bargetto et al. 2021)], and as such would be expected to be influenced similarly by extrinsic drivers and consequently to share model parameterization to some extent; this is exactly what we observe.

It seems notable that three of our four core/quasi‐core species share an identical model structure for *PENT* (Table 3). Reproductive seasons, interacting with year, are a dominant influence on *PENT* for three of these species; this interaction is replaced with one of year and rainfall only for *P. darwini* . Thus, more recruits are added to the population during the breeding season for three core species, and in good rainfall years for *P. darwini* .

Survival was strongly influenced by climatic seasonality (rainy/dry) in all three core rodent species. In other words, survival varies yearly but also differs across rainy seasons for these species, although these seasonal differences are minor and not all in the same direction; whereas mean *φ* was 12% lower in the wet season for *A. olivacea* , it was 3% and 7% higher for *P. darwini* and *O. degus* , respectively. For *T. elegans* , survival varied yearly, interacting with the reproductive season; mean ϕ was 24% lower in the breeding season. At a more arid site in the Andean foothills, annual rainfall was more fundamental to survival for *T. elegans* (Lima, Stenseth, et al. 2001), whereas seasonal and annual variation in rainfall was most influential for *P. darwini* (Crespin and Lima 2006; Lima, Julliard, et al. 2001).

Not surprisingly, given temporal variation in core parameters, the estimated number of recruits and population size both varied substantially across time, and as noted above, both were closely associated with rainfall, reflecting the trenchant nature of this extrinsic influence. One ancillary observation that emerges from analyses presented here is the value of CMR approaches relative to “traditional” estimates of population size that do not account for variation in trappability. One common approach is the MNKA (Hilborn et al. 1976; Krebs 1966), which estimates population size in a given census as the sum of unique individuals captured during that census plus any additional individuals captured both before and after this census, thereby assuming that these animals were present but not captured. We directly compared estimates of population size using a CMR approach against MNKA estimates for our species; for core species, CMR estimates were 2.8 ( *O. degus* ) to 5.9 ( *T. elegans* ) times higher than estimates based on MNKA, with extreme values as high as 210 times higher in some months for *P. darwini* . Linear regression of CMR estimates on MNKA estimates suggests a compelling linear relationship in some species ( *O. degus* ), but modest ( *A. olivacea* , *P. darwini* ) to very poor ( *T. elegans* ) relationships in other species characterized by low trappability. For those species that are well‐sampled with our monthly censuses, these differences likely indicate that MNKA does not adequately characterize population dynamics of these species and can potentially lead to incorrect inferences, presumably due to the inability of this metric to account for spatial or temporal variation in trappability. Of particular interest is whether these biases are consistent across biotic treatments, which is an issue we will be addressing in subsequent papers.

In general terms, these results agree with work conducted elsewhere on these species, although other studies have been based on much shorter time series. These other studies have emphasized the role of bottom‐up dynamics and intraspecific competition, and strong demographic responses to rainfall (Lima and Jaksic 1999b; Lima et al. 2006; Meserve, Kelt, et al. 2016; Previtali et al. 2009, and papers cited therein), although at least at Fray Jorge, rainfall appears to influence the two sigmodontine species differently, elevating carrying capacity for *P. darwini* but increasing the per capita growth rate in *A. olivacea* (Lima et al. 2006). At Las Chinchillas, *T. elegans* displays both seasonal (summer/fall > winter/spring) and interannual variation in trappability; recruitment is positively influenced by both population density and annual rainfall, and survival is negatively related to annual rainfall (Lima, Stenseth, et al. 2001). These authors argued that *T. elegans* populations at that site likely were food‐limited, which is in agreement with earlier work demonstrating direct density‐dependent feedbacks in that population (Lima and Jaksic 1998b, 1999b), and agrees with the available literature on all four core species.

### 4.2 Opportunistic Species Display Heterogeneous Model Structures

Whereas model parameterization for core species varied modestly, opportunistic species at Fray Jorge displayed more distinct model structures, which presumably reflect very different extrinsic drivers governing their demography (although we note that data limitations also constrained model complexity). Unlike core species, the evolutionary origins of opportunistic species are more diverse; *O. longicaudatus* appears to have entered Chile from Patagonian Argentina and then dispersed northward in Chile (Palma et al. 2005), whereas *A. longipilis* may have originated in central or north‐central Chile (Palma et al. 2010). Both species are more typical elements of mesic forested habitats, as noted elsewhere. *A. bennettii* likely originated in or near Norte Chico, but it is generally uncommon and patchily distributed where it occurs, and as with the other opportunistic species, it may be more restricted to mesic habitats than our core species. Additionally, whereas the demography of core species was well described by only one or at most two “competitive” models, opportunistic species yielded three or four models with ∆AICc < 2, presumably reflecting lower sample sizes or greater temporal heterogeneity; for example, we captured more individuals of the opportunistic *O. longicaudatus* and *A. longipilis* than we did of the core *T. elegans* , but whereas the latter was well described by a single best model, the former yielded three and four models, respectively (Table 3).

The resulting model structures are readily interpreted in terms of our understanding of the ecology of these species, all three of which appear to maintain refuge populations in aguadas and forests, located down‐ or upslope from our long‐term trapping grids, respectively. In particular, *A. bennettii* appears to favor aguada habitat, whereas *A. longipilis* tends to be more abundant in forests; *O. longicaudatus* is irruptive but overall similar in abundance in aguadas and forests at Fray Jorge (Milstead et al. 2007). As such, the thorn‐scrub habitat that characterizes our study plots likely is a secondary habitat for these species; supporting this, *A. longipilis* and *O. longicaudatus* have the highest evaporative water loss of ten species studied in Norte Chico (Cortés et al. 2000). These observations make sense in light of the model structures that emerged herein.

For *O. longicaudatus*, all three competitive models emphasized reproductive season, either additively (*p*) or interactively (*PENT*) with year, or alone or in combination with a trinary metric of annual rainfall (*φ*; Table 3). Trappability is modestly greater during the breeding seasons (0.359 vs. 0.319), but *PENT* and survival are 47% and 22% greater in the non‐breeding season. Hence, the available data support a hypothesis that this species enters our trappable population in the non‐breeding season (perhaps reflecting post‐breeding emigration of individuals from aguadas or forest habitat) and that they exhibit higher survival in this season as well. Very little work has been pursued on the demography of this species, and the most comprehensive efforts are from populations in the Valdivian rainforests of southern Chile, where it is one of four dominant species (Meserve et al. 1991). In primary growth temperate rainforest, numbers of this species are sporadic and irruptive, with low and highly variable survival rates (Meserve et al. 1991). Irruptions of this species have been attributed to cyclical masting of arborescent bamboos in this region (González et al. 2000; Murúa et al. 1996; Murúa et al. 1986; Sage et al. 2007), underscoring the central importance of intraspecific competition in this species (Murúa et al. 2003a). At Fray Jorge, we believe this species is limited by moisture and therefore an inhabitant primarily of aguada and bosque habitats. We have earlier argued for the presence of source‐sink processes in this species (Meserve et al. 1995) with irruptions to thorn scrub when rainfall leads to food production, releasing this species from food limitation, which likely regulates populations in southern Chile (Murúa et al. 2003a).

For *A. longipilis* , all three fundamental parameters (*p*, *PENT*, *φ*) were best modeled as functions of year plus reproductive season, although year plus climatic seasonality entered one of four competitive models for both *PENT* and *φ*, and year plus a binary metric of annual rainfall entered one model for *PENT*. Although we captured only 123 more individuals (24% more) relative to *A. bennettii* , total captures were five times greater, reflecting much higher trappability (Table 2). The general pattern, however, suggests that all three key parameters are strongly influenced by reproductive events, with rainfall a contributing factor to entry as well as survival. This matches field observations very well. As noted above, this species generally is more abundant in the fog forests at the top of the Altos de Talinay (Milstead et al. 2007). Our permanent sampling grids are located at the base of the Altos de Talinay, so the forests are as close as ca. 1 km from our grids, although movement may be facilitated by the use of ravines, which provide greater proximity to our grids. We infer these patterns to reflect demographic expansion in peripheral habitats under conditions of bottom‐up resource availability, followed by emigration from these peripheral habitats to our thorn‐scrub grids. In northern Chile, this species is largely insectivorous (Meserve 1981b), and the productivity of such prey in arid systems is strongly influenced by rainfall (Fuentes and Campusano 1985; Meserve, Vásquez, et al. 2016; Müller et al. 2024; Skendžić et al. 2021; van Dijk et al. 2024). Once animals reached our grids, survival (*φ*) was relatively high (Table 2) and about 4% greater in the non‐breeding season than during the breeding season. In the Valdivian temperate rainforest of southern Chile, this species appears to undergo regular ca. 3‐year cycles, albeit with relatively low amplitude, and is regulated largely by intraspecific competition (Murúa et al. 2003b). It is conceivable that such cyclicity may characterize *A. longipilis* populations in forest habitat at Fray Jorge, but within the thorn scrub, we believe that source‐sink dynamics (e.g., forest thorn scrub) likely dominate. Estimates of recruitment and population size (Figure 8c,d) illustrate the importance of rainfall and, as with *A. bennettii* , of temporal lags, with demographic pulses following rainy periods in the 1990s and early 2000s. Also notable, however, is the lack of such growth since about 2006. This has been a period of extended drought in north‐central Chile, with lower and less variable rainfall (Garreaud et al. 2020; Newman 2019), and we have noted strong impacts on the biota at our site (summarized in Armas et al. 2016).

**TABLE 2:** Summary of demographic parameters for seven small mammals at Fray Jorge, Chile.

	No. of Indiv. (MM/FF)	No. of captures	*p* (mean, range)	*PENT* (mean, range)	*φ* (mean, range)	Annual Survival	Births (mean, range)	*N* (mean, range)	Sex ratio (M:F)
Core									
*Abrothrix olivacea*	9773 (6455/3318)	24,135	0.633 (0.035–0.959)	2.992 × 10^−3^ (1.142 × 10^−4^–2.177 × 10^−2^)	0.660 (0.298–0.889)	6.847 × 10^−3^	8.807 (0.101–70.874)	30.215 (0.240–374.010)	2.03
*Phyllotis darwini*	9316 (5209/3107)	17,940	0.597 (1.115 × 10^−2^–0.972)	2.743 × 10^−3^ (1.035 × 10^−4^–9.651 × 10^−3^)	0.584 (0.265–0.742)	1.572 × 10^−3^	7.194 (0.077–26.286)	17.870 (0.708–54.796)	1.70
*Octodon degus*	6099 (3106/2993)	25,178	0.502 (0.011–0.965)	2.824 × 10^−3^ (1.296 × 10^−4^–1.105 × 10^−2^)	0.809 (0.430–0.930)	7.915 × 10^−2^	5.207 (3.153 × 10^−3^–20.731)	8.449 (0.186–32.547)	1.04
*Thylamys elegans*	1179 (712/467)	2437	0.416 (0.015–0.745)	2.870 × 10^−3^ (2.783 × 10^−4^–1.975 × 10^−2^)	0.682 (0.420–0.909)	1.017 × 10^−2^	1.337 (0.294–2.939)	4.987 (0.748–21.795)	1.55
Opportunistic									
*Oligoryzomys longicaudatus*	1782 (1207/575)	2596	0.338 (5.283 × 10^−3^–0.675)	2.569 × 10^−3^ (1.232 × 10^−4^–1.162 × 10^−2^)	0.536 (0.460–0.602)	5.622 × 10^−4^	3.867 (4.757 × 10^−3^–33.283)	9.169 (4.493 × 10^−3^–45.166)	2.15
*Abrothrix longipilis*	634 (407/227)	2996	0.649 (0.134–0.912)	3.028 × 10^−3^ (3.097 × 10^−4^–1.432 × 10^−2^)	0.768 (0.384–0.918)	4.210 × 10^−2^	0.623 (≈0–3.365)	3.267 (1.525 × 10^−4^–13.092)	1.86
*Abrocoma bennettii*	511 (240/271)	599	2.355 × 10^−2^ (1.094 × 10^−2^–5.353 × 10^−2^)	3.101 × 10^−3^ (0–1.170 × 10^−2^)	0.819 (0.680–0.950)	9.134 × 10^−2^	1.906 (2.337 × 10^−7^–8.372)	19.886 (1.153–70.771)	0.98

*Note:* The number of individuals and captures is the total from which all analyses are based. Trappability (*p*), survival (*φ*), annual survival (based on *φ*), and the probability of entry to the superpopulation (*PENT*) are taken from model‐averaging across all models, with the exception of *Oligoryzomys longicaudatus* , for which we model‐averaged the second through sixth models (see text). Births were derived from the top selected model (for *O. longicaudatus* , this was the second model; see text), and population size (*N*) was model‐averaged for all species other than *Octodon*, for which the top selected model was used. Finally, the sex ratio is taken from estimates of *N* for males and females.

Finally, for *A. bennettii* , all three basic parameters were best modeled as functions of reproductive season and a tripartite metric of annual rainfall, either interactively (*p*) or additively (*PENT*, *φ*). As noted above, however, data limitations for this species precluded more complex models, so these results do not represent a subset from a larger pool of potential model structures. Given this, we interpret parameter characteristics but emphasize caution in making too much of these results. Trappability and especially *PENT* remain low at all times for this species, whereas survival (*φ*) is generally quite high (Table 2). However, reflecting the former two parameters, we caution that the demographic parameters that are derived from these values (e.g., recruitment and estimated *N*) need to be interpreted cautiously. Indeed, this species illustrates the challenges associated with estimating values when *p* and *PENT* are low. Whereas the estimated values for recruitment appear reasonable for many years (Figure 9c), those for population size frequently are exceedingly unrealistic (Figure 9d). We assume, nonetheless, that these reflect temporal patterns for this species and illustrate the influence of rainfall on this species (Figure 9c,d). Unlike other species, both recruitment and population size in *A. bennettii* appeared to exhibit a lag of up to 2 years relative to annual rainfall. A hystricomorph rodent, *A. bennettii* shares a relatively K‐selected reproductive strategy with *O. degus* , and prolonged gestation may contribute to this delay. Additionally, however, this may suggest that populations in core (non‐thorn scrub) habitats saturate during rainy periods, and as conditions dry out, we see dispersal of animals seeking more favorable habitat, a pattern similar to the “saturation dispersal” that Lidicker Jr. (1975) proposed for California Voles ( *Microtus californicus* ). *A. bennettii* would be a compelling subject for management‐relevant life‐history work and is large enough to support radiotransmitters that could provide detailed information on their ecology and life history. Literature on the demography of this species is quite limited (Muñoz‐Pedreros and Gil 2009; Patton and Emmons 2015), so any effort would likely yield a substantial advancement.

### 4.3 Temporal and Inter‐Specific Variation in Trappability

Trappability is a critical metric in CMR analyses, as it reflects the likelihood that a given animal will be captured in a survey, given that it is alive and in the sampled area. Low trappability leads to poor precision in parameter estimates, which is reflected in large SE estimates and broad confidence limits. It also influences how much the observed number of captures is adjusted to produce an estimated population size. Trappability varied strongly over time for most species: for two species ( *P. darwini* and *O. degu*; Table 3), virtually all well‐supported models included a time (or an interaction between year and season) effect on *p*, suggesting that this parameter varied across sampling occasions (every month of every year of sampling). For three other species, all well‐supported models included an additive and/or interactive effect of year and reproductive season (Table 3). These results suggest high temporal variability in capture probability. The only exception was that the top model structure for *p* for an opportunistic species ( *A. bennettii* ) included an effect of season based on rainfall; this was a consequence of small sample size rather than a lack of temporal variability in *p*. Using data from the first 18 years of this study (Mar. 1989—May 2007), Previtali et al. (2010) found that trappability for *O. degus* was best modeled as an additive function of climatic season (winter/spring vs. summer/fall) and year, and while they used a different analytical approach and bimonthly encounters, they also demonstrated strong year‐ and season‐specific variation in *p*.

**TABLE 3:** Model selection results for seven small mammals at Bosque Fray Jorge National Park, Chile, testing for the effect of different extrinsic factors on trappability (*p*), the probability of new animals entering the population (*PENT*), survival (*ϕ*), and the superpopulation size (*N*).

Model no.	*p*	*PENT*	Phi (*φ*)	*N*	npar	AICc	∆AICc	*w*_*i*_	Deviance
*Abrothrix olivacea* (Core species)									
1	yr * repro_season + sex	yr * repro_season	yr * rain_season	Sex	189	51923.833	0	0.997	−71633.005
2	yr * repro_season	yr * repro_season	yr * rain_season	Sex	188	51935.805	11.973	0.003	−71618.999
3	yr * repro_season + sex	yr * repro_season	yr * repro_season	Sex	189	52110.202	186.369	0	−71446.636
4	yr * repro_season	yr * repro_season	yr * repro_season	Sex	188	52121.497	197.665	0	−71433.307
5	yr * repro_season + sex	yr * repro_season	yr + rain_season	Sex	159	52157.040	233.208	0	−71338.904
*Phyllotis darwini* (Core species)									
1	time	yr * rain_season	yr * rain_season	Sex	490	37042.473	0	1	−74908.146
2	time	yr * rain_season	yr * repro_season	Sex	490	37090.742	48.269	3.300 × 10^−11^	−74859.877
3	time	yr * rain_season	yr + rain_season	Sex	460	37096.605	54.132	1.759 × 10^−12^	−74790.585
4	time	yr * rain_season	yr + repro_season	Sex	460	37117.600	75.127	0	−74769.589
5	time	yr * rain_season	yr + wet_dry	Sex	460	37120.895	78.422	0	−74766.294
*Octodon degu* (Core species)									
1	time	yr * repro_season	yr * rain_season	1	473	59513.796	0	0.666	−24332.067
2	time	yr * repro_season	yr * rain_season	Sex	474	59515.178	1.383	0.334	−24332.763
3	time	yr * repro_season	yr * repro_season	1	473	59602.296	88.500	0	−24243.566
4	time	yr * repro_season	yr * repro_season	Sex	474	59604.220	90.425	0	−24243.720
5	time	yr * repro_season	yr + rain_season	1	443	59702.721	188.925	0	−24080.858
*Thylamys elegans* (Core species)									
1	yr * repro_season	yr * repro_season	yr + repro_season	Sex	158	7889.685	0	0.759	−6230.136
2	yr * repro_season + sex	yr * repro_season	yr + repro_season	Sex	159	7891.982	2.298	0.241	−6230.136
3	yr * repro_season + sex	yr * repro_season	yr + repro_season	1	158	7907.693	18.008	9.330 × 10^−5^	−6212.127
4	yr * repro_season	yr * repro_season	yr * repro_season	Sex	188	7909.849	20.165	3.174 × 10^−5^	−6279.829
5	yr * repro_season + sex	yr * repro_season	yr * repro_season	Sex	189	7912.209	22.524	9.756 × 10^−6^	−6279.832
*Oligoryzomys longicaudatus* (Opportunistic species)									
2	yr + repro_season	yr * repro_season	repro_season	Sex	94	6347.765	0.000	0.250	−12838.590
3	yr + repro_season	yr * repro_season	annual_ppt_LMH + repro_season	Sex	96	6347.777	0.012	0.249	−12842.899
4	yr + repro_season	yr * repro_season	annual_ppt_LMH * repro_season	Sex	98	6349.039	1.274	0.132	−12845.965
5	yr	yr * repro_season	annual_ppt_LMH + repro_season	Sex	95	6352.004	4.239	0.030	−12836.510
6	yr	yr * repro_season	repro_season	Sex	93	6352.775	5.010	0.020	−12831.423
*Abrothrix longipilis* (Opportunistic species)									
1	yr + repro_season	yr + repro_season	yr + repro_season	Sex	95	7196.066	0	0.287	55.032
2	yr + repro_season	yr + rain_season	yr + repro_season	Sex	95	7197.373	1.307	0.150	56.339
3	yr + repro_season	yr + wet_dry	yr + repro_season	Sex	95	7197.761	1.695	0.123	56.727
4	yr + repro_season	yr + repro_season	yr + rain_season	Sex	95	7198.047	1.981	0.107	57.013
5	yr + repro_season	yr + rain_season	yr + rain_season	Sex	95	7199.443	3.377	0.053	58.409
*Abrocoma bennettii* (Opportunistic species)									
1	repro_season * annual_ppt_LMH	repro_season + annual_ppt_LMH	repro_season + annual_ppt_LMH	1	15	2455.057	0	0.363	−2470.909
2	repro_season * annual_ppt_LMH	repro_season + annual_ppt_LMH	repro_season + annual_ppt_LMH	Sex	16	2456.167	1.110	0.208	−2471.911
3	repro_season * annual_ppt_LMH	repro_season + annual_ppt_LMH	repro_season * annual_ppt_LMH	Sex	18	2456.476	1.419	0.179	−2475.850
4	repro_season * annual_ppt_LMH	repro_season + annual_ppt_LMH	repro_season * annual_ppt_LMH	1	17	2458.470	3.412	0.066	−2471.729
5	repro_season * annual_ppt_LMH	repro_season * annual_ppt_LMH	repro_season + annual_ppt_LMH	1	17	2458.790	3.732	0.056	−2471.409

*Note:* Presented are the top five models for each species, with the exception of *Oligoryzomys longicaudatus* , for which we model‐averaged the second through sixth models (see text). Species are listed in the order presented in the text (core species followed by opportunistic species).

### 4.4 Population Size Is Driven by the Number of Recruits

**FIGURE A3:**
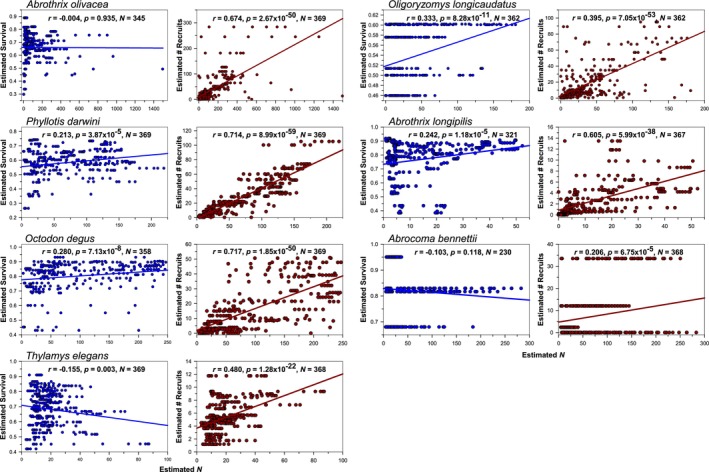
Correlation of estimated survival (blue) and estimated number of recruits (dark red) on estimated population size for four core species (left) and three Opportunistic species (right) at Bosque Fray Jorge National Park.

Across all species studied, the estimated population size is influenced more by the number of recruits than by survival. Correlations of recruits with population size are positive and highly significant for all species. Correlations of ϕ with population size are lower for all species, functionally flat for one species ( *A. olivacea* ), and weakly negative for two (Appendix Figure A3). The slopes of the relationship between recruits and population size are 1.2–152 times greater than those of ϕ on population size; interestingly, this difference is greater for all core species than for opportunistic species, suggesting that even for those species for which the thorn‐scrub habitat is secondary habitat, arrival to these plots, or in situ recruitment, has a greater influence on population size than does survival. The species for which the two slopes are most similar is *O. longicaudatus* ; this species is well known for irruptive population dynamics (Meserve et al. 1991; Meserve et al. 1995; Murúa et al. 2003a; Murúa et al. 1986); we suspect that the greater similarity among slopes for this species may reflect the fact that population expansion in Fray Jorge occurs at times of abundant rainfall, which provides more amenable habitat for this species, leading to higher survival probabilities. For all other species, the slope of recruits on population size was at least 2.5× steeper than that for ϕ on population size. As noted above, we interpret these relationships with caution for *A. bennettii* , for which limited data make estimates *p*, *PENT*, ϕ and population size particularly imprecise.

### 4.5 Population and Community Dynamics in an ENSO‐Dominated Ecosystem

Overlaying a historical influence of ENSO dynamics in this region is the growing impact of climate change and anthropogenic desertification. Although the damage caused by centuries of subsistence agriculture has left the region's biota stressed (Bahre 1979; Kelt and Meserve 2016; Squeo et al. 2016), recent climate change and an ongoing “mega‐drought” (Garreaud et al. 2020; Newman 2019) have further challenged the biota here. Elsewhere, we have shown that prolonged drought reduces both survival and reproduction by *Octodon* (Previtali et al. 2010), but these impacts evidently are even more substantial for the shorter‐lived sigmodontine rodents, as *Octodon* rapidly assumed local dominance in terms of proportional biomass during the prolonged drought that initiated ca. 2003 (Meserve, Kelt, et al. 2016). During such extended periods of drought, foraging by *Octodon* led to indirect facilitation of invasive ephemeral plants (Jiménez et al. 2011). Only continued monitoring will document the extent to which such changes are reversible, or whether they may be, or portend, a critical transition in this system (Scheffer 2009).

This study contributes to a growing literature demonstrating the overwhelming influence of rainfall on small mammals of arid and semiarid regions, and complements earlier work in Chile that focused on local extinction/colonization processes, intrinsic versus extrinsic factors driving fluctuations, and feedback structure in time‐series data, emphasizing *P. darwini* but with lesser emphasis on *A. olivacea* and *T. elegans* (Crespin and Lima 2006; Lima and Jaksic 1998a, 1999b, 1999c; Lima, Julliard, et al. 2001; Lima et al. 1996; Lima et al. 1998; Lima et al. 2002; Lima, Stenseth, et al. 2001). In spite of the overwhelming influence of rainfall in this system, both competition (inter‐ and intraspecific) and predation do play roles. Predation has an ambiguous influence on foraging (Kelt et al. 2004; Yunger et al. 2002), and whereas initial analyses of data on control and predator‐exclusion grids at Fray Jorge suggested that predators suppressed *Octodon* numbers and survival (Meserve et al. 1993; Meserve et al. 1996), subsequent work suggested that first‐order processes (intraspecific competition) were more important than predation for both *O. degus* and *P. darwini* (Previtali et al. 2009). Nonetheless, *O. degus* appears less restricted to “safe” habitat beneath shrubs where predators have been excluded (Lagos et al. 1995) and they spend more time on vigilance activities in open habitat than among shrubs (Vasquez et al. 2002). Hence, it may be that predation has more immediate, short‐term behavioral influences on at least some species at this site, whereas density dependence, driven by bottom‐up forcing in response to rainfall, is more important over longer time periods. We look forward to addressing such questions in future analyses.

Importantly, both the extent (30 years) and intensity (4 consecutive nights/month) of our data make the analyses presented here unique in South America, and rare at a global scale for arid or semiarid regions (Hayes et al. 2017; Kelt 2011). Moreover, our application of the superpopulation CMR modeling approach to these long‐term data advances our knowledge of these species in at least three specific areas. First, in a system notable for temporal variation in resources (and hence in trappability), this method provides estimates of abundance, survival, and recruitment while accounting for imperfect or spatiotemporally variable detection (trappability). Second, we show that recruitment varies much more widely than survival, and this variation is the primary driver of population dynamics of our core study species. Third, we clarify how nearly all core species respond rapidly to seasonal and annual variation in rainfall patterns, whereas opportunistic species exhibit different responses to seasonal or annual variation in rainfall, which we attribute to their preference for non‐thorn‐scrub habitat.

Finally, this study provides the first demographic insight for nearly the entire small mammal community in our study site, and for all seven species, we apply modern analytical tools to the longest time series of CMR data available. As such, observations reported herein provide initial foundations for understanding the demography of *A. bennettii* as well as *A. longipilis* and *O. longicaudatus* in semiarid environments, and the most data‐rich characterizations of the four core species. Where these results mirror those from other studies, they provide important reinforcement of our understanding of the biology of these species; where they differ, results presented herein bolster ecological understanding and contribute importantly to resource management, as well as to the biology of relatively short‐lived mammals in highly seasonal environments. Our study site, a national park and UNESCO Biosphere Reserve, is one of the very few protected areas in Norte Chico, and continued anthropization of the surrounding landscape risks increasing the isolation of populations here. Just as vegetative communities of this park “are the best available model for ecological restoration projects in this region of Chile” (Squeo et al. 2016, 12), we believe that the vertebrate community provides a benchmark against which other sites in similar habitat should be assessed. In a world confronting climate change and anthropogenic desertification, the observations reported herein are increasingly valuable.

## Author Contributions

**Douglas A. Kelt:** conceptualization (equal), formal analysis (equal), funding acquisition (equal), investigation (equal), methodology (equal), project administration (equal), resources (equal), software (equal), supervision (equal), visualization (equal), writing – original draft (lead). **Peter L. Meserve:** conceptualization (lead), funding acquisition (equal), investigation (equal), methodology (lead), project administration (equal), resources (equal), supervision (equal), visualization (equal), writing – review and editing (supporting). **Alejandra J. Troncoso:** project administration (supporting), supervision (supporting). **W. Bryan Milstead:** data curation (lead), investigation (equal), methodology (equal), writing – review and editing (supporting). **M. Andrea Previtali:** data curation (equal), investigation (supporting), methodology (supporting). **Julio R. Gutiérrez:** conceptualization (equal), funding acquisition (equal), investigation (equal), methodology (supporting), project administration (equal), supervision (equal). **Madan K. Oli:** conceptualization (lead), formal analysis (lead), methodology (lead), software (lead), writing – original draft (supporting), writing – review and editing (equal).

## Conflicts of Interest

The authors declare no conflicts of interest.

## Data Availability

R code and data are provided at figshare at: https://figshare.com/s/cf634e2c9e10d8855da3.

## Appendix A

### A.1

**TABLE A1:** Preliminary modeling to establish the best parameterization for trappability (*p*); *p* was allowed to vary as a function of reproductive season, year, dry or wet years, additive and interactive effects of these variables, and, when data permitted, time. *N* was modeled as constant or sex‐specific, and model structures for *PENT* and *φ* were allowed to be fairly general (this varied across species, depending on data availability).

*p*	*PENT*	Phi (*φ*)	*N*	npar	AICc	DeltaAICc	weight	Deviance
*Abrothrix olivacea* (Core species)								
Time	yr + season	yr	Sex	415	52110.651	0	0.670	−71909.959
Time	yr + season	yr + season	Sex	416	52112.204	1.552	0.308	−71910.479
Time	yr	yr	Sex	414	52118.355	7.703	0.014	−71900.184
Time	yr	yr + season	Sex	415	52119.527	8.876	0.008	−71901.084
Time	yr + season	1	Sex	385	52475.313	364.662	4.371 × 10^−80^	−71483.218
*Phyllotis darwini* (Core species)								
Time	yr + season	yr + season	Sex	430	37564.507	0	0.000	−74259.477
Time	yr + season	yr	Sex	429	37565.723	1.216	0.002	−74256.158
Time	yr	yr + season	Sex	429	37587.168	22.661	2.84 × 10^−2^	−74234.713
Time	yr	yr	Sex	428	37587.490	22.983	2.88 × 10^−2^	−74232.288
Time	yr + season	1	Sex	399	37675.704	111.197	1.39 × 10^−1^	−74083.203
*Octodon degus* (Core species)								
Time	yr + season	yr	1	413	60428.902	0	0.451	−23292.548
Time	yr + season	yr	Sex	414	60430.184	1.282	0.238	−23293.335
Time	yr + season	yr + season	1	414	60430.224	1.322	0.233	−23293.295
Time	yr + season	yr + season	Sex	415	60432.421	3.519	0.078	−23293.165
Time	yr	yr	1	412	60497.937	69.035	8.66 × 10^−2^	−23221.444
*Thylamys elegans* (Core species)								
yr * season	yr	yr + season	Sex	127	7957.5639	0	0.440	−6092.0329
yr * season	yr + season	yr + season	Sex	128	7958.3025	0.739	0.304	−6093.5294
yr * season + sex	yr	yr + season	Sex	128	7959.6889	2.125	0.152	−6092.1429
yr * season + sex	yr + season	yr + season	Sex	129	7960.4847	2.921	0.102	−6093.5841
yr * season + sex	yr	yr + season	1	127	7972.0712	14.507	0.000	−6077.5256
*Oligoryzomys longicaudatus* (Opportunistic species)								
yr + season	yr + season	yr + season	Sex	94	6375.566	0	1.000	−12810.789
yr + season	yr + season	season	Sex	64	6397.582	22.015	1.660 × 10^−5^	−12724.808
yr + season	yr + season	yr	Sex	93	6399.680	24.114	5.800 × 10^−6^	−12784.517
yr	yr + season	yr + season	Sex	93	6406.008	30.442	2.450 × 10^−7^	−12778.190
yr + season	yr + season	1	Sex	63	6408.320	32.754	7.720 × 10^−8^	−12711.964
*Abrothrix longipilis* (Opportunistic species)								
yr + season + sex	yr	yr + season	Sex	95	7188.879	0	0.403	47.845
yr + season + sex	yr + season	yr + season	Sex	96	7189.090	0.211	0.362	45.913
yr + season + sex	yr + season	yr	Sex	95	7191.582	2.704	0.104	50.548
yr + season + sex	yr	yr	Sex	94	7191.683	2.804	0.099	52.790
yr + season	yr	yr + season	Sex	94	7195.620	6.741	0.014	56.727
*Abrocoma bennettii* (Opportunistic species)								
Season	yr + season	1	1	36	1904.374	0	0.648	−3067.559
Season	yr + season	1	Sex	37	1905.615	1.241	0.348	−3068.594
1	yr + season	1	1	35	1916.119	11.745	0.002	−3053.546
1	yr + season	1	Sex	36	1917.360	12.987	0.001	−3054.572
Sex	yr + season	1	1	36	1917.4548	13.081	0.001	−3054.478
